# HPTN 062: A Pilot Randomized Controlled Trial Exploring the Effect of a Motivational-Interviewing Intervention on Sexual Behavior among Individuals with Acute HIV Infection in Lilongwe, Malawi

**DOI:** 10.1371/journal.pone.0124452

**Published:** 2015-05-11

**Authors:** Audrey Pettifor, Amy Corneli, Gift Kamanga, Kevin McKenna, Nora E. Rosenberg, Xuesong Yu, San-San Ou, Cecilia Massa, Patricia Wiyo, Diana Lynn, Jenae Tharaldson, Carol Golin, Irving Hoffman, HPTN 062 Study Protocol Team

**Affiliations:** 1 University of North Carolina at Chapel Hill, NC, United States of America; 2 FHI 360; Durham, NC, United States of America; 3 UNC Project Lilongwe, Lilongwe, Malawi; 4 The Statistical Center for HIV/AIDS Research & Prevention, Seattle, WA, United States of America; Johns Hopkins University, UNITED STATES

## Abstract

**Objective:**

We pilot tested a Motivational Interviewing (MI) –based counseling intervention for individuals with Acute HIV Infection (AHI) to reduce risky sexual behavior in Lilongwe, Malawi.

**Methods:**

Twenty-eight individuals diagnosed with AHI were randomized to receive either brief education alone, or the brief education plus the MI-based intervention, called Uphungu Wanga. Participants in Uphungu Wanga received four sessions delivered on the day of diagnosis, three days later and at weeks 1 and 2 with a booster session at week 8; participants were followed for 24 weeks from diagnosis. An interviewer administered quantitative questionnaire was conducted at baseline and at weeks 2, 4, 8, 12, 16, 20 and 24. Semi-structured qualitative interviews (SSI) were conducted at weeks 2, 8, 12, and 24.

**Results:**

The majority of participants in both arms reported rapid and sustained behavior change following diagnosis with AHI. Very few participants reported having sex without a condom after diagnosis. Participants reported a trend towards fewer sex partners and abstaining from sex during study follow-up. Participants in the MI-based arm provided concrete examples of risk reduction strategies in the SSIs while those in the brief education arm primarily described reducing risk behavior, suggesting that the MI-based group may have acquired more risk reduction skills.

**Conclusions:**

Individuals in both study arms reduced risky sexual behaviors after diagnosis with AHI. We found few major differences between study arms during the 6-month follow up period in self-reported sexual behaviors therefore a MI-based intervention may not be needed to trigger behavior change following AHI. However, comparing the MI-based intervention to repeated brief education sessions made it difficult to assess the potential benefit of an MI-based intervention in a setting where standard counseling often consists of one post-test session. Nevertheless, provision of counseling immediately following diagnosis with HIV to support behavior change should remain a priority.

**Trial Registration:**

ClinicalTrials.gov NCT01197027

## Introduction

Acute HIV Infection (AHI) is the 3–4 weeks immediately following transmission when an individual remains HIV sero-negative with a detectable viral load. During this period and up to 24–26 weeks following transmission the viral load reaches remarkably high levels and results in extreme infectiousness [1, 2]. Mathematical modeling studies have indicated that, despite its brief duration, the high-level of infectiousness of AHI and difficulties detecting it likely lead to a substantial proportion of new HIV infections, even in high prevalence settings [2, 3]. Estimates using empirical data from Lilongwe, Malawi show that AHI may account for 38% of all new infections in this setting characterized by a mature epidemic where 10% of the adult population is estimated to be HIV-infected [2].

Studies of HIV-infected individuals, including those diagnosed with AHI, have shown that, while many people reduce risky sexual activities after diagnosis, others continue to engage in sexual activity and some continue to engage in unprotected sex with multiple partners [4–7]. Given that AHI occurs for a relatively short period of time, avoiding onward transmission during this period requires early diagnosis with rapid behavior change. However, little is currently known about whether individuals diagnosed with AHI will change their sexual behavior, particularly whether a positive prevention intervention could facilitate this change [8].

Research conducted in Lilongwe, Malawi and Johannesburg, South Africa documented that, while the majority of participants with AHI reported being able to abstain or use condoms after AHI diagnosis, a significant minority continued to engage in unprotected sex [4]. For these individuals, significant challenges to behavior change included initiating condom use with main/significant partners when condom use was not previously the norm, and lack of disclosure of their sero-status to these partners. In response to the need to develop a risk reduction intervention during the period of AHI, we pilot-tested a counseling intervention, based in Motivational Interviewing (MI), to reduce the risk of HIV transmission during this highly infectious period. We hypothesized that individuals who received the MI intervention would report fewer unprotected sex acts than those not receiving the intervention. In this manuscript, we explore whether the MI intervention resulted in a reduction in risky behavior, primarily, unprotected sex acts. This analysis was part of a larger study that included determining the feasibility and acceptability of the intervention [9].

## Methods

### The protocol for this trial and supporting CONSORT checklist are available as supporting information; see S1 CONSORT Checklist and S1 Protocol.

**Fig 1:**
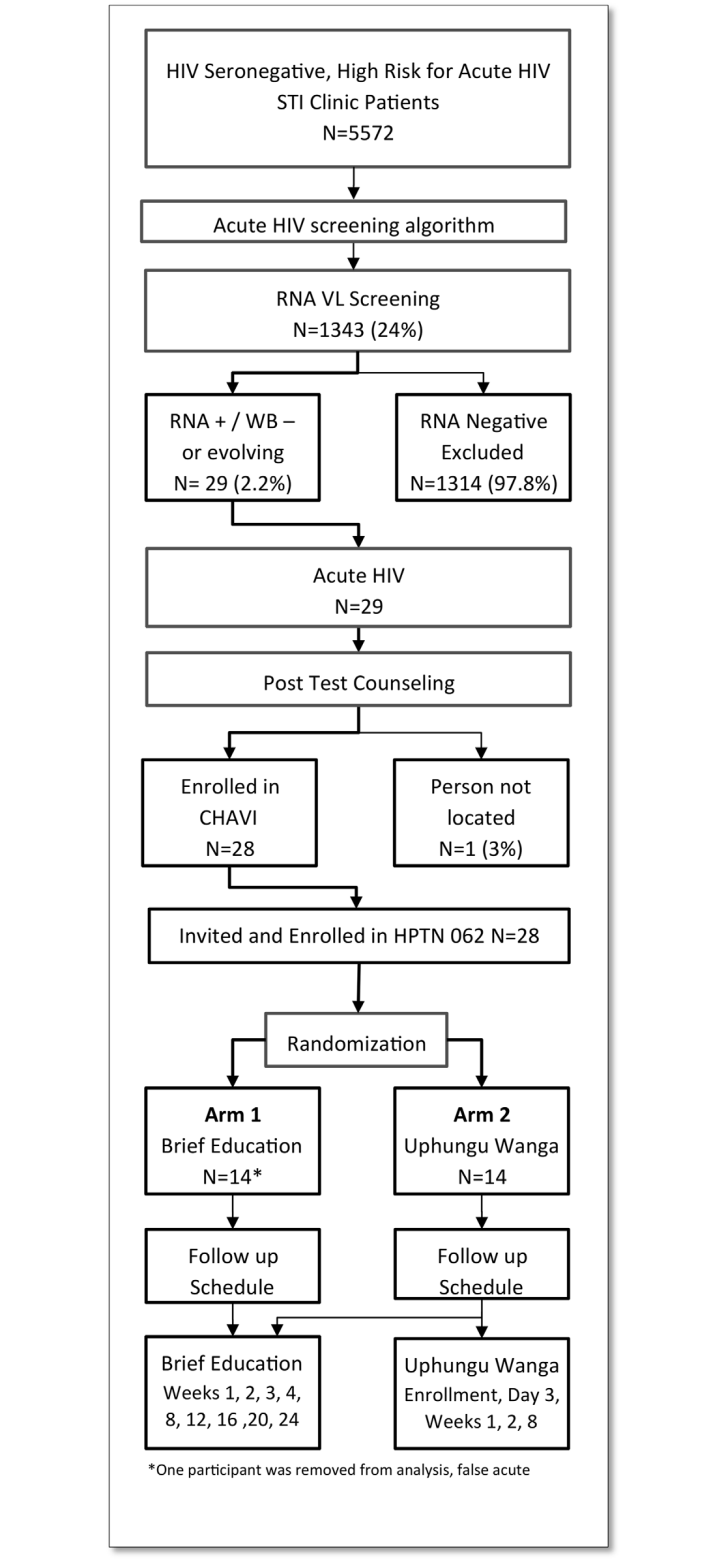
Study Flow for HPTN 062, Lilongwe, Malawi.

HIV Prevention Trials Network (HPTN) 062 was a single-site, two-arm, randomized pilot intervention study conducted in Lilongwe, Malawi (clinicaltrials.gov registration number NCT01197027, the full trial protocol can be accessed at www.HPTN.org)[9]. Participants were recruited from CHAVI 001, a study to identify and follow individuals with AHI. Individuals diagnosed with AHI who provided informed consent were block-randomized in a 1:1 ratio to either brief education about HIV and AHI, provided as part of CHAVI 001, or the same brief-education plus MI-based counseling intervention called “Uphungu Wanga” (translated as “my own counseling”)[9] (see Fig 1). A blocked randomization design with 11 blocks of size 4 and 1 block of size 2 without stratification was used at the study site using a central computerized system.

Participants in both arms were followed for 24 weeks at weeks 1, 2, 3, 4, 8, 12, 16, 20, and 24, which coincided with the CHAVI 001 clinic visit schedule, and received the brief education at each visit (see Corneli A et al. for AHI messages provided)[9]. Participants assigned to the Uphungu Wanga arm also received four additional MI sessions plus a booster at the following times: on the day of AHI diagnosis, three days later, and at weeks 1, 2, and 8 post-diagnosis (booster). At each study visit, participants in Uphungu Wanga completed all CHAVI 001 procedures before going to a separate location on the same hospital campus to proceed with the Uphungu Wanga counseling.

The Uphungu Wanga intervention was informed by formative research conducted with CHAVI 001 participants [4] as well as by the Options Project [10] and Project SafeTalk [11–13]—two interventions demonstrated to be effective at reducing risky sexual behaviors among people living with HIV/AIDS. The Information, Motivation, Behavioral Skills (IMB) model [14] provided a framework for the Uphungu Wanga counseling, and the sessions were delivered using an MI-based approach, a client-centered approach to counseling [15, 16]. Previous interventions that were informed by the IMB model and MI have demonstrated a reduction in sexual risk behaviors among people living with HIV—hence we chose the IMB model and MI for our intervention [10, 13, 17, 18]. At each session, counselors tailored counseling using a standardized counseling guide; they assessed a participant’s knowledge of AHI and gave additional explanation as needed, assessed a participant’s motivation for behavior change, and assessed a participant’s self-efficacy to adopt their chosen risk reduction strategy. Given the short duration of AHI, the intervention needed to be delivered in a short period of time immediately after diagnosis and focus on short-term behavior change goals (e.g., a few days or weeks). Three core components formed the foundation of the Uphungu Wanga intervention and were the focus of counseling sessions: 1) understanding AHI, 2) enhancing clients’ intrinsic motivation to practice abstinence or 100% condom use during anal and vaginal sex throughout the period of AHI, and 3) disclosing HIV status to current partners. At each session participants set a goal specific to safer sex.

Three counselors participated in 5 days of training—facilitated by MI experts and informed by a step-by-step training manual—on MI counseling techniques and on the use of standardized session guides to deliver the intervention. One of two trained female counselors facilitated most Uphungu Wanga sessions; for two participants, one trained male counselor facilitated all 5 of their sessions. To help the counselors maintain fidelity to the protocol, they used quick reference guides—which outlined the key intervention steps of each session—during the counseling session.

### Study Participants

To be eligible, participants had to be age 18 years or older and to have acute or early HIV infection. AHI, per CHAVI 001 protocol, was defined as having parallel sero-negative results with a positive nucleic acid test (NAT). Early HIV infection was defined as having a positive or discordant parallel serologic result, a positive NAT, and an evolving, negative, or indeterminate Western Blot. Participants were enrolled between February 2010 and December 2011 and the last follow up visit was in April of 2012. The protocol aimed to enroll up to 46 individuals however only 28 individuals were enrolled during the study period due to a lower than anticipated incidence rate.

### Ethics Statement

The National Health Science Research Committee in Malawi and the Protection of Human Subjects Committee (PHSC) at FHI 360 in the U.S. approved the study. The Institutional Review Board at the University of North Carolina at Chapel Hill provided ongoing review of the study but deferred approval authority to the PHSC. Written informed consent was obtained from all participants, the consent form and process was approved by both human subject review committees. The study was registered with clinicaltrials.gov in September 2010, after patient enrollment began. The delay occurred because the study was registered with the NIH Division of AIDS regulatory support center prior to participant enrollment, trials that were not for drugs or devices were not required to register at the time, and trials that are not for serious or life threatening conditions can take place within one year of enactment. The authors confirm that all ongoing and related trials for this drug/intervention are registered.

### Measures and Data Collection

Interviewers administered a quantitative questionnaire to all participants at baseline and at weeks 2, 4, 8, 12, 16, 20 and 24. The questionnaire assessed information about: 1) the number of sex partners in the past 3 months (baseline only) and in the past 2 weeks (all visits) 2) number of anal and vaginal sex acts (reported separately) in the past 2 weeks; 3) number of unprotected anal and vaginal sex acts (reported separately) in the past 2 weeks; and 4) whether unprotected anal and vaginal sex acts were with partners with an HIV-positive, HIV-negative, or unknown status. Interviewers also obtained detailed partner-specific information about each partner the participant had sex with in the past 3 months (baseline) and in the past 2 weeks (follow-up visits) using a partner grid. For each partner identified by a participant, data were captured regarding the type of partner (e.g., spouse, casual partner, sex worker), whether a condom was used during the most recent sex act, consistency of condom use with partner (never, rarely, sometimes, frequently, always), if the partner had other partners, typical frequency of sex (daily, 3–6 times/week, 1–2 times/week, 2 or 3 times a month, once a month or less, 1 time only), whether the partner is HIV-infected and if so, if the partner is on ART.

Interviewers also conducted semi-structured interviews (SSIs) with participants in both arms at weeks 2, 8, 12, and 24. Data on sexual behaviors emerged from responses to questions asked to [1] participants in the Uphungu Wanga arm about their experiences in attempting to achieve their sexual behavior goal they had identified at each session, including any facilitators or difficulties faced in trying to reach their goals and their perceived importance of and confidence in performing safer sex behaviors described in their goal and 2) participants in both arms about the acceptability (e.g. perception of the content and perceived effectiveness) of the brief education or Uphungu Wanga counseling. Data on sexual behaviors also emerged from participants’ responses to questions designed request them to compare and contrast their sexual behavior before and after their AHI diagnosis, describe any changes in their sexual behavior over time after their AHI diagnosis and the reasons for these changes, and to describe the impact the AHI messages had on their sexual behaviors.

### Analyses

At baseline, descriptive statistics of characteristics of study participants are presented by intervention arm. At baseline and follow-up the number and proportion of participants reporting each behavior within each intervention arm are presented. Wilcoxon rank sum test was used to compare the differences between arms for count data, and Wilcoxon signed rank test was used to compare the differences over time for count data. Fisher’s exact test was used to compare the differences in proportions between arms, and McNemar test was used to compare the differences in proportions between follow-up vs. baseline.

We developed a case summary for each participant that described their sexual behaviors and the context surrounding those behaviors using data from the qualitative SSIs conducted at weeks 2, 4, 8, and 12. Each summary included the participant’s demographic information (i.e., gender, age, marital status, education, occupation); a description of sexual behaviors before AHI diagnosis; and a description of current sexual behaviors and the context surrounding those behaviors, including any changes and the reasons for those changes, as described at each interview. For participants in the Uphungu Wanga arm, the summaries also included participants’ descriptions of their attempts to reach their individual safe sex goals and any facilitators or challenges they faced in attempting safer sex behaviors. Case studies were reviewed to better understand the sexual behavior context (e.g. relationship status, factors associated with risk behavior, context of safer sexual behavior) for each individual participant. Next we conducted thematic inductive analysis among the case studies from participants in both study arms to identify overall themes in the topics described above. We used data reduction to identify the most prominent themes within each topic and their frequencies, followed by a comprehensive data summary with illustrative quotes.

Sample size is limited by the expected low incidence rate for AHI. This study is not powered to evaluate the efficacy of the intervention effect. However, if the effectiveness of the intervention is high, a sample size of 27 would yield 87% power to detect a difference of 2 in the mean number of unprotected sex acts when the mean number of unprotected sex acts is 0.5 in the intervention arm and 2.5 in the control arm. The power was estimated using a Monte Carlo simulation of 10, 000 trials under the assumption that the number of unprotected sex acts follows a negative binomial distribution with dispersion parameter equal to 2. The Wilcoxon Rank Sum test was used for comparison of number of sex acts in two arms with a Type I error rate of 0.05.

## Results

Twenty-eight individuals were enrolled and randomized to receive either the standard brief education counseling alone, or the brief education plus the Uphungu Wanga intervention counseling. One participant in the brief education arm was discontinued from the study because of a false AHI diagnosis resulting in 13 participants in the brief education arm and 14 in the Uphungu Wanga arm (n = 27). More women, individuals aged 18–24 years, and more individuals who reported being married were enrolled in the Uphungu Wanga arm than the brief education arm although the differences were not statistically significant (Table 1).

**Table 1:** Characteristics of Study Participants at Baseline in HPTN 062

	Brief Education (BE)		Uphungu Wanga (UW)	
	N = 13		N = 14	
Gender				
Male	10	(77%)	8	(57%)
Female	3	(23%)	6	(43%)
Age				
18–24	2	(15%)	6	(43%)
25–29	6	(46%)	6	(43%)
30–40	5	(38%)	2	(14%)
Education				
≤ primary completed	6	(46%)	8	(57%)
some secondary	3	(23%)	3	(21%)
≥ secondary completed	4	(31%)	3	(21%)
Marital Status				
Never married	6	(46%)	4	(29%)
Married	3	(23%)	6	(43%)
Divorced/separated	4	(31%)	4	(29%)
Partners in the last 3 months*				
1	6	(46%)	8	(62%)
≥2	7	(54%)	5	(38%)
Partners in the last 2 weeks				
0	5	(38%)	6	(43%)
1	6	(46%)	6	(43%)
2	2	(15%)	2	(14%)
Sex acts in last 2 weeks				
0	5	(38%)	6	(43%)
1–3	6	(46%)	4	(29%)
≥4	2	(16%)	4	(29%)
Any unprotected sex in last 2 weeks				
No	6	(46%)	9	(64%)
Yes	7	(54%)	5	(36%)
Alcohol consumption in <2 weeks				
0 days	9	(69%)	8	(57%)
1–4 days	4	(31%)	4	(29%)
> = 5 days	0	(0%)	2	(14%)
Alcohol consumption before sex in <2 weeks				
No	12	(92%)	11	(79%)
Yes	1	(8%)	3	(21%)

All sex acts are vaginal sex acts. No anal sex was reported over the duration of the study.

*Missing data

A large proportion of individuals in both arms reported engaging in unprotected sex in the weeks and months before diagnosis with AHI (Tables 1, 2 and 3). At baseline, the median number of sex partners in the past 2 weeks was 1 in both arms, while at almost all follow up visits the median number of sex partners in both arms was 0 (Table 2). The median number of sex acts in the past 2 weeks at baseline was 1 in both arms, while at follow up it was 0 at most visits in both arms (except for week 8 and 24 in the Uphungu Wanga arm) (Table 2). At baseline, 54% percent of participants in the brief education arm and 36% of participants in the Uphungu Wanga arm reported sex without a condom in the past 2 weeks, while at follow up, no participant in either arm reported sex without a condom except for 1 Uphungu Wanga participant at week 8 and 1 participant in the brief education arm at week 12 (Table 2). In the brief education arm, 13 participants at baseline reported having 24 sexual partners in the last three months and reported never using condoms with 46% of these partners. Among participants in the Uphungu Wanga arm, 12 reported 19 sexual partners in the last three months at baseline and reported never using condoms with 47% of these partners (Table 3).

**Table 2:** Sexual Behavior in the Last Two Weeks at Baseline and Follow-Up.

	Number of Sexual Partners in the Last 2 Weeks					Number of Sex Acts in the last 2 Weeks					Proportion Reporting Unprotected Sex				
	BE (N = 13)	UW (N = 14)	Overall	p-value (between arms)	p-value (over time)	BE (N = 13)	UW (N = 14)	Overall	p-value (between arms)	p-value (over time)	BE	UW	Overall	p-value (between arms)	p-value (over time)
**Baseline**	1 (0, 1)	1 (0, 1)	1 (0, 1)	0.2	Reference	1 (0, 2)	1 (0, 5)	1 (0, 3)	0.7	Reference	54%	36%	44%	0.3	Reference
**week 2**	0 (0, 0)	0 (0, 1)	0 (0, 1)	0.07	0.04	0 (0, 0)	0 (0, 2)	0 (0, 1)	0.07	0.1	0%	0%	0%	1.0	0.001
**week 4**	0 (0, 0)	0 (0, 1)	0 (0, 1)	0.3	0.09	0 (0, 0)	0 (0, 2)	0 (0, 1)	0.3	0.05	0%	0%	0%	1.0	0.001
**week 8**	0, (0, 0.5)	1 (0, 1)	0 (0, 1)	0.2	0.4	0 (0, 0.5)	1 (0, 2)	0 (0, 2)	0.1	0.3	0%	7%	4%	1.0	0.002
**week 12**	0 (0, 1)	0 (0, 1)	0 (0, 1)	0.6	0.06	0 (0, 1.5)	0 (0, 2)	0 (0, 2)	0.8	0.4	8%	0%	4%	0.5	0.002
**week 16**	0 (0, 0)	0 (0, 1)	0 (0, 1)	0.4	0.03	0 (0, 0)	0 (0, 1)	0 (0, 1)	0.4	0.2	0%	0%	0%	1.0	0.001
**week 20**	0 (0, 1)	0 (0, 1)	0 (0, 1)	1.0	0.08	0 (0, 1)	0 (0, 2.5)	0 (0, 1.5)	0.6	0.2	0%	0%	0%	1.0	0.001
**week 24**	0 (0, 1)	1 (0, 1)	1 (0, 1)	0.2	0.8	0 (0, 2)	2 (0, 3)	1 (0, 3)	0.09	0.7	0%	0%	0%	1.0	0.004

Table 2 displays medians with interquartile ranges for the number of sexual partners, number of sexual acts, and proportion of persons reporting any unprotected sex in the last two weeks at each time point. Wilcoxon rank sum exact tests were used to compare the differences between arms. Wilcoxon signed rank exact tests were used to compare the differences over time.

**Table 3:** Characteristics of Participants' Sexual Partners at Baseline and Post-Intervention.

	Baseline		Week 4		Week 8		Week 24	
	BE	UW	BE	UW	BE	UW	BE	UW
	N = 13	N = 12	N = 2	N = 5	N = 3	N = 8	N = 5	N = 9
Attributes	(N = 24 partners)	(N = 19 partners)	(N = 3 partners)	(N = 7 partners)	(N = 5 partners)	(N = 9 partners)	(N = 6 partners)	(N = 10 partners)
Partnership type								
Spouse	3 (13%)	6 (32%)	1 (33%)	3 (43%)	2 (40%)	5 (63%)	1 (17%)	6 (60%)
Girl/Boyfriend	5 (21%)	5 (26%)	0 (0%)	2 (29%)	0 (0%)	1 (13%)	3 (50%)	2 (20%)
Casual partner	10 (42%)	6 (32%)	2 (67%)	2 (29%)	3 (60%)	2 (25%)	1 (17%)	0 (0%)
CSW/client	6 (25%)	1 (5%)	0 (0%)	0 (0%)	0 (0%)	0 (0%)	1 (17%)	2 (20%)
Other	0 (0%)	1 (5%)	0 (0%)	0 (0%)	0 (0%)	0 (0%)	0 (0%)	0 (0%)
Has partner had other partners?								
Yes	14 (58%)	9 (47%)	0 (0%)	2 (29%)	0 (0%)	1 (11%)	0 (0%)	1 (10%)
Possibly	3 (13%)	4 (21%)	1 (33%)	5 (71%)	1 (20%)	6 (67%)	3 (50%)	6 (60%)
No	7 (29%)	6 (32%)	2 (67%)	0 (0%)	4 (80%)	2 (22%)	3 (50%)	3 (30%)
How often do you have sex?								
≥3 times/week	3 (13%)	4 (21%)	2 (67%)	1 (14%)	2 (40%)	2 (22%)	2 (33%)	3 (30%)
1–2 times/week	7 (29%)	8 (42%)	0 (0%)	5 (71%)	0 (0%)	3 (33%)	3 (50%)	5 (50%)
<1 time/week	10 (42%)	4 (21%)	1 (33%)	1 (14%)	3 (60%)	4 (44%)	1 (17%)	2 (20%)
1 time only	4 (16%)	3 (16%)	0 (0%)	0 (0%)	0 (0%)	0 (0%)	0 (0%)	0 (0%)
How often do you use condoms								
Always	5 (21%)	2 (11%)	3 (100%)	7 (100%)	5 (100%)	8 (89%)	6 (100%)	10 (100%)
Sometimes	8 (33%)	8 (42%)	0 (0%)	0 (0%)	0 (0%)	1 (11%)	0 (0%)	0 (0%)
Never	11 (46%)	9 (47%)	0 (0%)	0 (0%)	0 (0%)	0 (0%)	0 (0%)	0 (0%)
Partner's HIV Status								
HIV-positive	0 (0%)	1 (5%)	1 (33%)	2 (40%)	0 (0%)	1 (13%)	1 (20%)	0 (0%)
HIV-negative	1 (4%)	1 (5%)	0 (0%)	0 (0%)	1 (25%)	1 (13%)	1 (20%)	1 (17%)
Unknown	23 (96%)	17 (90%)	2 (67%)	3 (60%)	3 (75%)	6 (75%)	3 (60%)	5 (83%)

At each time point, participants were asked about characteristics of their partners. Partner characteristics are presented in this table. At most time points some participants did not report any partners. For this reason, the number of participants declines after baseline. Some participants report more than one partner. For this reason the number of partners exceeds the number of participants at most time points.

No social harms were reported by either study arm.

Data from the quantitative surveys thus documented changes in sexual behavior after diagnosis with AHI. Participants in both arms reported using condoms more often and there was a trend towards reducing partner numbers after diagnosis compared to before diagnosis. Below we provide more contextual information from the qualitative SSIs on behavior change that participants reported as a result of the participating in the study.

### Abstinence

In both study arms, many participants reported not having a sex partner after AHI diagnosis (short term abstinence during the AHI period was one of the goals offered in the Uphungu Wanga arm). In the SSIs, many participants reported that their sex partners had been away since the participants’ AHI diagnosis due to work (migrant partners) or schooling in other locations and therefore they had been abstaining from sex for this reason. Some participants also said they had been abstaining due to having no opportunity to engage in sex because a partner was away or because they had no partner but they would have sex if there was an opportunity:

***Since I was diagnosed with HIV I have been abstaining and when I want to have sex I use a condom*. *(27 year-old unmarried male*, *brief education arm)***

Other participants reported more actively abstaining, that is, choosing not to have sex despite having an opportunity to do so. Participants also discussed that while they were able to abstain during the period of AHI, they would have protected sex in the future when they have a sex partner or get married:

***Yes*, *I have achieved Abstinence*!*[as a result of the intervention]… when I find a partner in future*, *I may choose to use condoms*. *(20 year-old divorced female*, *Uphungu Wanga arm)***

Participants who were married or in long term relationships discussed the challenge of abstaining, even for a short period of time. One individual reported how the concept of abstinence was counter to that of marriage:

***Interviewer*: *When you say abstinence will make marriage not to work well*. *What do you mean*?**

***Participant*: *You know this is marriage*, *when you want to be helped [have sex] you are supposed to call each other for you to be helped*. *Now when it does not go that way*, *thoughts come in to say why am I staying here [marriage] then*? *Then let me just leave*! *Is it marriage anymore when we are not having sex*? *(28 year-old married male*, *Uphungu Wanga arm)***

Some participants talked specifically about how the Uphungu Wanga counseling helped them adhere to their goal of abstaining during the study.

***So when my madam [wife] was away*, *there were some people who would say*, *‘Is your madam gone*?*’ Mostly*, *it was girls*, *yah*? *‘How come you are staying alone*?*’ But because I had made up my mind about my goal*, *and that such ways cannot help us—there times when I think about my HIV status and how I can protect myself*. *So from that time up to now*, *I have not encountered any problem*. *I have not taken advantage of my wife’s absence for me to engage myself in other behaviors*. *I can see that the encouragement that I am receiving here and even today*, *has helped me a lot*. *(26 year old married male*, *Uphungu Wanga arm)***

A few participants in both arms described a loss of desire to have sex after AHI diagnosis and explained that abstaining from sex was therefore not difficult for them.

### Alternatives to Sex

Participants in the Uphungu Wanga arm, but not the brief education arm, described other means of satisfying their sexual desires through non-penetrative sex (a method suggested as an option in the Uphungu Wanga counseling). Six participants mentioned masturbation as a means that they could use to satisfy their sexual desire without having to have sex.

***… when you feel like having sex*, *you can fulfilled your desire without actually having physical contact with a woman… You can start thinking of someone you had sex with sometime back and the desire can be fulfilled*, *sometimes you can masturbate and that’s the end*. *The feeling is the same (26 year-old married male*, *Uphungu Wanga arm)***

### Condom Use

In the quantitative questionnaire, the majority of participants who engaged in sex after their AHI diagnosis reported having sex only with their main/regular partners and the frequency of sex remained fairly constant (Table 3). Moreover, after diagnosis, the vast majority of participants who reported having sex reported having sex using a condom. In fact, only two reports of sex without a condom occurred over the 24 weeks of follow-up (Table 2). The first participant was in the Uphungu Wanga arm and reported having had sex without a condom at the week 8 visit. She was married and she described in an SSI that her husband was very opposed to using a male condom. Through discussions with a counselor, she described in the SSIs finding alternatives to safer sex through the use of female condoms:

***These things were indeed difficult*, *you know*, *condoms are like plastics*, *yah*, *we were used to having plain sex*. *So especially on the part of my husband*, *he was adamant saying that he cannot use the condom*, *because he said he did not feel anything*. *But because I was afraid that if I can have plain sex with him*, *I may infect him*, *I just had to put up with it*, *but my husband kept on saying he had no feeling with a condom… Well*, *this time around*, *he has been able to cooperate because I told him that it was either that or I was ready to leave*, *but also with the female condoms… after I had presented my problem on condom use with my partner to my counselor*, *I was advised to try the female condoms*. *This helped me because I never knew anything about female condoms*. *Now I am able to use these condoms and they are of great help*. *(28 year-old married female*, *Uphungu Wanga arm)***

The second participant was in the brief education arm and reported in the quantitative questionnaire having had sex without a condom in the last two weeks at the week 12 visit. He was unmarried and reported having many sex partners prior to his AHI diagnosis. Although he said he reduced the number of overall partners and generally used condoms with his partners after his AHI diagnosis, he described in an SSI that he had sex with a woman without using a condom on one occasion after he became drunk.

Other participants also described challenges with using condoms in their SSIs. Some described the difficulty of introducing condoms suddenly into a relationship when they had not been used previously and a prior lack of knowledge on how to use condoms correctly.

***… at first I used to worry that I may not be able to use condoms properly—using the recommended methods of use effectively*, *I thought that maybe I may not be able to manage*. *But I have seen that these are doable now I have managed and now I am able to use effectively*. *(20 year-old divorced female*, *Uphungu Wanga arm)***

Participants talked during the SSIs about the importance of counseling in motivating them to use condoms. For example, participants described that disclosure was an important part of being able to implement safer sex behaviors, including condom use (disclosure was an explicit goal of the third Uphungu Wanga session).

***From the first day that I started to get counseling*, *is the day I started using the condoms—I see that I have not encountered any problems; I accepted this and have disclosed to my wife about my HIV status and she is also satisfied with it*. *So I do not find it a problem using the condom…My partner supported me very much*, *this is because she accepted what I had told her that*, *“this had just happened but I love my life*! *So it is difficult for me to have intimate relationships with you without having to protect myself*!*” Well she now understand*, *there are also times when at bedtime*, *she would have the condoms ready*. *This is because she has accepted what I have told her*. *(26 year-old married male*, *Uphungu Wanga arm)***

Being empowered to be able to correctly use condoms with their partners was also described as important by some participants (both amrs focused on demonstrating correct condom use skills and allowing participants to practice putting condoms on models).

***I was very successful because with what I am learning and what I am doing out there I am able to see that it is acceptable*. *I find myself using condoms all the time I am having sex*. *Every time I ask a sex partner to have sex I make sure a condom is available*. *I don’t see it as strange so through the counseling session I have learned that using a condom is very important*. *(26 year old married male*, *Uphungu Wanga arm)***

Although participants in the Uphungu Wanga arm provided more specific details about how counseling motivated them to use condoms, provided solutions to challenges with using condoms, and helped them devise plans to achieve their goals to use condoms, participants in the brief education arm also described in their SSIs how counseling helped them realize the importance of using condoms.

***Interviewer*: *Can you tell me more about when you talk of condom use being challenging*, *enslaving and yet at the same time you have told me that you are using condoms regularly now*?**

***Participant*: *Yes*, *I will tell you*. *It is sometime back when I thought that condoms were not good*, *but now I have realized that condoms are important*. *I now use the condom because I know that it is very important—it is the shield of my life*. *(35 year-old divorced male*, *brief education arm)***

### Partner Number Reduction

In both arms, participants reported in the quantitative interview fewer partners post-diagnosis compared the time prior to diagnosis with AHI. At baseline, all participants reported at least one partner in the past three months and nearly half reported at least two partners (range 2–4). In contrast, at follow up the majority of participants reported no sex partners in the previous 2 weeks (Table 2). In the SSIs, participants in both arms also talked about reducing partners after diagnosis of AHI:

***They are different now because now I have only one partner while during that time*, *when I had [acquired] acute HIV [before diagnosis]*, *I had a lot of partners*. *(20 year-old divorced female*, *Uphungu Wanga arm)***

After diagnosis, a greater proportion of partners were reported in the quantitative questionnaire to be main partners or boyfriend/girlfriends rather than casual partners or sex workers (Table 3). Participants also reported having fewer partners that they perceived to have other sexual partners after diagnosis than prior to diagnosis (Table 3).

### Alcohol Use

Many participants talked about the association between drinking alcohol and having unprotected sex with casual partners. At baseline, 30% of participants reported in the quantitative questionnaire drinking alcohol 1–4 days in the last two weeks, 7% reported drinking alcohol more than five days in the last two weeks, and 15% reported having had unprotected sex when drinking in the prior 2 weeks (Table 1). Also at baseline, 6 people in the Uphungu Wanga arm reported drinking alcohol in the last two weeks compared to 2 people at weeks 8 and 24. Four people in the brief education arm reported drinking at baseline and this did not change in week 8 and 24 (data not shown). Data from the SSIs also stressed the importance of alcohol contributing to risk behavior.

***Interviewer*: *what do you think is the link between heavy drinking behavior and sexual behavior*?**

***Participant*: *There is a big link because if I go for beer drinking in a bar*, *there are women in the bar who help in cutting the beer*. *[Interviewer note*: *as previously stated in the other IDIs*, *this participant likes chibuku; this is a type of beer which comes in packets and not bottles*. *The bargirls are the ones who help in cutting the packets as a matter of good service]*. *Because you are drunk and what happens in the bar*, *you lose your senses and you are taken up by the women*, *you call for a woman*, *a prostitute you discuss and have sex with her*. *(28 year-old separated male*, *brief education arm)***

Participants in both arms talked about acknowledging the role of alcohol in increasing risk behavior.

***And when I am drunk I was confused*, *up to the point of having sex without protection…*.*Just doing things because I am drunk*. *So now I have thought of stopping drinking beer because such things can bring death… When they tested me [PW*: *Tested for HIV] they told me to come back*, *when I came they told me that I had acute HIV*. *So the counseling that they gave me is the one that encouraged me to change*. *I know that I contracted this HIV because I was doing things when I was drunk*. *(20 year-old separated female*, *brief education arm)***

Participants in the Uphungu Wanga arm in particular discussed the importance of counseling in identifying alcohol as a trigger that resulted in risky behavior and helping them to stop drinking and thus reduce risky behavior. Some participants in Uphungu Wanga specifically focused on avoiding alcohol venues as one of their goals.

***Or maybe going to entertainment centers*, *especially when you consider having intoxicating drinks*, *these can cause someone to be influenced and have sex with a woman*. *So during our sessions we discussed with the counselor here and I feel that whenever I go out to entertainment centers*, *I do not take long in these places*. *I just spend little time with friends and then leave*. *(23 year-old unmarried male*, *Uphungu Wanga arm)***

### Re-Infection

Participants in both arms mentioned avoiding re-infection with HIV as a motivation for abstaining from sex, using condoms and reducing partner numbers. As described by these two participants:

***Interviewer*: *What has made you change*?**

***Participant*: *I have changed because I have seen that there is a chance for me to re-infect myself with a strain of HIV which may be difficult to treat or to transmit to others*. *(26 year-old married male*, *Uphungu Wanga arm)***

***Participant*: *I knew that when the virus has just entered the body*, *this the time you spread and re-infect yourself and if you re-infect yourself*, *the immune system get weaker and I chose to prevent to avoid adding more infections*. *I knew ways of preventing*. *It is good to abstain*. *(28 year-old married male*, *Uphungu Wanga arm)***.

## Discussion

We found the majority of participants reported rapid and sustained behavior change for the 24 weeks following diagnosis with AHI. All but one participant in each arm of the study reported no unprotected sex acts during study follow-up and most reported fewer sex partners (in particular casual sex partners).

We observed no major differences in reported sexual behaviors between study arms. Importantly, both arms of the study received an intensive follow-up schedule after AHI diagnosis which resulted in being seen by a clinician on the day of diagnosis and at weeks 1, 2, 4, 8, 16 and 24 after diagnosis. Previous research has found that the first year following any HIV diagnosis can be a critical time for interventions as individuals struggle to change behavior and deal emotionally with their disease [19, 20]. In most settings, the standard of care following diagnosis with HIV (if not eligible for anti-retroviral therapy (ART)) involves one post-test HIV counseling session on the day of diagnosis. With the roll out of ART, individuals who are eligible for treatment are often instructed to come back to the clinic for a number of different visits before starting ART to give them time to adjust to the illness and diagnosis. Individuals who are not eligible for ART are often instructed to come back at some point in the future (e.g., at 6 months) to check their CD4 count. Many individuals not initially eligible for ART, however, are lost to care after diagnosis with HIV [21]. Our findings suggest that visits with a clinician and/or counselor in the first few weeks after diagnosis with AHI are feasible and acceptable [9] and also may result in behavior change. Further, being diagnosed with HIV presents a “teachable moment” when one is more likely to be motivated to establish and/or maintain good self-care and risk reduction [22]. This teachable moment may be even more powerful in the setting of AHI where one can likely identify exactly when individuals acquired the virus and what they did to acquire it. Taking advantage of this highly teachable moment is likely to increase the effectiveness of behavioral interventions, especially for individuals with AHI [22].

At baseline, many of the participants reported having casual partners in the three months before diagnosis with AHI. Many of the participants did not have stable partners at baseline and instead reported having sex with multiple casual partners during the time when they became infected with HIV. After diagnosis most participants reported reducing partner numbers, particularly casual partners. For participants who were not with stable partners, many chose to abstain and found this to be feasible in the short term, particularity among participants reporting having partners who were travelling or not living with them in Lilongwe. While having a travelling partner may have put these individuals at risk of acquiring HIV (either through their partner’s behaviors or their own), it also seemed to facilitate abstaining from sex during the period of AHI. Given the short duration of AHI, the focus of this intervention was on rapid behavior change for a brief, finite period of time. The goal of the intervention was to reduce risk behavior during this acute, highly infectious phase of infection—not necessarily for the rest of the individual’s life. A number of participants commented that some of the changes they had made following diagnosis, in particular abstinence, would likely not be sustained in the long term. There will be a need for different types of behavior change interventions for individuals at different phases of the disease stage as individuals face different types of challenges.

Alcohol misuse was widely reported in the SSIs, especially leading up to and immediately before AHI diagnosis. Participants talked about the role of drinking at bars and ‘entertainment’ venues where they would drink too much and then have sex with one or more partners. While the importance of alcohol misuse has been identified as an important risk factor for HIV in sub-Saharan Africa, identifying this link among those with AHI temporally in relation to when they acquired HIV provides even stronger evidence of a causal relationship. Moreover, to date there have been few rigorously evaluated interventions aimed at addressing risky alcohol consumption to reduce HIV risk in sub-Saharan Africa [23, 24]. A number of participants, particularly in the Uphungu Wanga arm, reported avoiding drinking venues as a means of reducing their HIV risk during AHI because they identified drinking and the venues themselves as triggers to risky sexual behavior—identifying triggers of sexual risk behavior was a component of the Uphungu Wanga intervention. In a setting such as Lilongwe, despite having a generalized epidemic, it appears that many new infections may be occurring in such venues, and interventions need to target not only drinking venues, but also their clientele and staff.

Despite observing similar reported behaviors between arms, there were notable differences between arms from the qualitative interviews. Participants in the Uphungu Wanga arm provided concrete examples of how they changed behavior and the skills they used to reduce risk—something that was not often described in the interviews with participants in the brief education arm. Individuals in the Uphungu Wanga arm also talked about the importance of having goals, which mostly focused on abstinence, condom use or avoidance of risk, in motivating them to reduce risk. They also talked about triggers they identified for risk behavior and steps they took to avoid such triggers (for example, avoiding drinking venues) and alternatives to having sex, such as masturbation. Individuals in the Uphungu Wanga arm also described talking with their counselors about challenges they were having with safer sex goals and reviewing alternative strategies that helped them overcome these challenges. While participants in the brief education arm also described practicing safer sex, they did not give such concrete examples of how this was accomplished compared to participants in the Uphungu Wanga arm, and instead primarily focused on expressing their belief that it was important to protect themselves from re-infection or their partners from HIV. Given the potential for social desirability bias in participants’ responses to an interviewer’s questions in a study such as this, the details described by participants in the Uphungu Wanga arm strengthen the evidence that behavior change was in fact attempted, and such evidence perhaps gives more credibility to the premise that behavior change was attempted and achieved by participants in the Uphungu Wanga arm more so when compared to participants in the brief education arm.

### Limitations

The first limitation is that the main outcomes of the paper are based on self-reported, sensitive behaviors which are subject to social desirability, particularly in the context of unblinded risk reduction intervention. Very few participants in either arm reported engaging in unprotected sex, making it difficult to assess the impact of the intervention on this outcome. Methods to improve reporting of sensitive behaviors are particularly important in behavioral interventions among HIV positive people where biomarkers of risk are not readily available. Importantly, given the difficulty of finding individuals with AHI, this pilot was conducted among participants who were part of an intensive observational study of AHI, CHAVI 001, which involved a number of clinic visits and blood draws. Given that the intervention took place in this context, and that the Uphungu Wanga intervention and assessments took place after the CHAVI visits, it is difficult to assess the effect of the Uphungu Wanga intervention on its own. Third, although this study was exploratory in nature and thus not designed to have power to detect changes between arms, the small sample size prevented us from having sufficient statistical power to detect differences between arms. In addition, due to the small sample size, we could not control for differences in risk behaviors and marital status between arms at baseline, making comparisons over time more difficult.

## Conclusion

Overall, we found that individuals diagnosed with AHI rapidly reduced risky sexual behavior and sustained safer behaviors following intensive clinical follow-up and counseling in the first few months following diagnosis with acute HIV in Lilongwe, Malawi. We found few major differences between study arms during the 6-month follow up period after diagnosis in AHI in self-reported sexual behaviors, suggesting that perhaps an MI-based intervention may not be needed to trigger behavior change during the short duration of AHI. However, the intensive control arm in this study made it difficult to assess whether an MI-based intervention would be better than a one time post-test counseling session, which is the standard of care in most settings. Qualitative data suggests that participants in the MI-based arm may have acquired more skills to reduce risk behavior however there were no differences reported in sexual behaviors between arms. Future interventions for individuals diagnosed with both acute and chronic HIV should prioritize engaging individuals in the time period following diagnosis with HIV to offer support and encourage behavior change, including adherence to ART for those eligible, and to maximize the teachable moment that diagnosis offers.

## Supporting Information

S1 Consort Checklist CONSORT checklist. (DOC)

S1 Protocol Trial protocol. (PDF)
