# Supplementary material for: HPTN 062: A Pilot Randomized Controlled Trial Exploring the Effect of a Motivational-Interviewing Intervention on Sexual Behavior among Individuals with Acute HIV Infection in Lilongwe, Malawi
Source: PLoS One. 2015 May 11;10(5):e0124452. doi: 10.1371/journal.pone.0124452 (PMC4427322; doi:10.1371/journal.pone.0124452)
Supplement: S1 Protocol — (PDF) [file pone.0124452.s002.pdf]

HPTN 062
Feasibility and Acceptability Study of an Individual-Level Behavioral Intervention for Individuals with Acute and Early HIV-Infection
DAIDS ID: 10667

A Study of the HIV Prevention Trials Network

Non-IND Study

Sponsored by:

US National Institutes of Health:
US National Institute of Allergy and Infectious Diseases
US National Institute on Drug Abuse
US National Institute of Mental Health

Protocol Co-Chair:

Audrey Pettifor PhD, MPH
University of North Carolina at Chapel Hill
Chapel Hill, North Carolina, USA

Protocol Co-Chair:

Amy Corneli PhD, MPH, CHES
Family Health International
Durham, North Carolina USA

FINAL Version 2.0
10 September 2009

HPTN 062
Feasibility and Acceptability Study of an Individual-Level Behavioral Intervention for Individuals with Acute and Early HIV-Infection

TABLE OF CONTENTS

LIST OF ABBREVIATIONS AND ACRONYMS....iii
PROTOCOL TEAM ROSTER....iv
PROTOCOL SUMMARY....1
1.0 INTRODUCTION....3
1.1 Background....3
1.2 Preliminary Studies....5
1.3 Study Rationale....9
2.0 STUDY OBJECTIVES AND DESIGN....10
2.1 Primary Objectives....10
2.2 Secondary Objectives....10
2.3 Study Design....10
3.0 STUDY POPULATION....12
3.1 Inclusion Criteria....13
3.2 Recruitment Process....13
3.3 Participant Randomization....13
3.4 Participant Retention....13
3.5 Participant Withdrawal....14
4.0 BEHAVIORAL INTERVENTIONS....14
4.1 Experimental Enhanced Individual Counseling....14
4.2 Standard Risk Reduction Counseling....20
4.3 Standardization and Quality Control....20
5.0 STUDY PROCEDURES....21
5.1 Screening/Enrollment (Day 0)....21
5.2 Follow-up Visits at Weeks 1, 2, 4 and 8....22
5.3 Follow-up Visits at Weeks 12, 16, 20 and 24....22
5.4 Language of Interview and Translation Process....23
5.5 Methods to Improve Self-Reported Sexual Behavior....23
6.0 ADVERSE EVENTS AND SOCIAL HARMS....23
7.0 STATISTICAL CONSIDERATIONS....24
7.1 General Design....24
7.2 Study Endpoints....24
7.3 Sample Size and Accrual....26
7.4 Randomization....29
7.5 Blinding....29
7.6 Analysis Plan....29
8.0 HUMAN SUBJECTS CONSIDERATIONS....32
8.1 Ethical Review....32
8.2 Informed Consent....32
8.3 Risks....33
8.4 Benefits....34
8.5 Incentives....34

8.6 Confidentiality 35
8.7 Study Discontinuation 35
9.0 LABORATORY SPECIMENS AND BIOHAZARD CONTAINMENT 35
10.0 ADMINISTRATIVE PROCEDURES 36
10.1 Study Activation 36
10.2 Study Coordination 36
10.3 Study Monitoring 36
10.4 Protocol Compliance 37
10.5 Investigator's Records 37
10.6 Use of Information and Publications 37
11.0 REFERENCES 38
APPENDICES 40
Appendix I - Schedule of Visits and Procedures 41
Appendix IIA - Sample Participant Enrollment Consent Form 42
Appendix IIB - Sample Counselor Consent Form 47

LIST OF ABBREVIATIONS AND ACRONYMS

AHI	acute HIV-infection
AIDS	acquired immunodeficiency syndrome
ART	antiretroviral therapy
CHAVI	Center for HIV/AIDS Vaccine Immunology
CORE	(HPTN) Coordinating and Operations Center
CRF	case report form
DAIDS	Division of AIDS
EC	Ethics Committee
EIA	enzyme immunoassay
HIV	human immunodeficiency virus
HPTN	HIV Prevention Trials Network
IMB	Information, Motivation, Behavioral Skills Model
IRB	Institutional Review Board
NAT	nucleic acid test
NIAID	(US) National Institute of Allergy and Infectious Diseases
NIDA	(US) National Institute on Drug Abuse
NIH	(US) National Institutes of Health
NIMH	(US) National Institute of Mental Health
OHRP	Office for Human Research Protections
RNA	ribonucleic acid
SDMC	(HPTN) Statistical and Data Management Center
SCHARP	Statistical Center for HIV/AIDS Research & Prevention
SSP	study-specific procedures
US	United States

HPTN 062
Feasibility and Acceptability Study of an Individual-Level Behavioral Intervention for Individuals with Acute and Early HIV-Infection

PROTOCOL TEAM ROSTER

Chiwoza Bandewa
College of Medicine-Johns Hopkins Project
P.O. Box 1131 Chipatala Avenue
Blantrye, Malawi
Phone: 265-8-205-816
Email: **cbandawe@gmail.com**

David Burns, MD
National Institute of Allergy and Infectious Diseases, DAIDS PSB
6700 B Rockledge Drive
Room 5249, MSC 7628
Bethesda, MD 20892-7628 USA
Phone: 1-301-435-8896
Fax: 1-301-402-3684
E-mail: **burnsda@niaid.nih.gov**

Tom Coates, PhD
University of California, Los Angeles,
David Geffen School of Medicine, Box 957320
Department of Medicine
Division of Infectious Disease
10940 Wilshire Boulevard, Suite 1220
Los Angeles, CA 90024 USA
Phone: 1-310-794-3580
Fax: 1-310-470-6768
E-mail: **tcoates@mednet.ucla.edu**

Myron S. Cohen, MD
University of North Carolina Chapel Hill
547 Burnett Womack, CB #7030
Chapel Hill, NC 27514 USA
Phone: 1-919-966-2536
Fax: 1-919-966-6714
E-mail: **mscohen@med.unc.edu**

Amy Corneli, PhD, MPH, CHES
Family Health International
2224 E NC Highway 54
Durham, NC 27713
Research Triangle Park, NC 27709 USA
Phone: 1-919-544-7040
Fax: 1-919-544-7261
E-mail: **acorneli@fhi.org**

Vanessa Cummings
HPTN NL QA/QC Coordinator
Johns Hopkins Univ. Dept of Pathology
Pathology Building 306
600 N Wolfe Street
Baltimore, MD 21287 USA
Phone: 1-410-502-5296
Fax: 1-410-614-6739
E-mail: **vcummin1@jhmi.edu**

Deborah Donnell, PhD
SCHARP-FHCRC
1730 Minor Ave N. MP-1195
PO Box 19024
Seattle, WA 98109-1024 USA
Phone: 1-206-667-5661
Fax: 1-206-667-6888
E-mail: **deborah@scharp.org**

Lynda Emel, PhD
FHCRC-SCHARP
1100 Fairview Ave N., LE-400
PO Box 19024
Seattle, WA 98109-1024 USA
Phone: 1-206-667-5803
Fax: 1-206-667-4812
E-mail: **lemel@scharp.fhcrc.org**

Andrew Forsyth, PhD
National Institute of Mental Health
6001 Executive Boulevard
Location: NSC - Neuro Science Center, 6204
Rockville, MD USA
Phone: 1-301-443-8403
Fax: 1-301-443-9719
E-mail: **af183p@nih.gov**

Carol Golin, MD
University of North Carolina
Center for AIDS Research
135C UNC Sheps Center for Health Services Research
725 Martin Luther King Boulevard--CB7590
Chapel Hill, NC 27599-7590 USA
Phone: 1-919-966-7939
Fax : 1-919-966-3811
E-mail: **Carol_Golin@unc.edu**

Deborah Hilgenberg, MPH
Family Health International
2224 E NC Highway 54
Durham, NC 27713
Research Triangle Park, NC 27709 USA
Phone: 1-919-544-7040
Cell: 1- 919-451-1061
Fax: 1-919-544-0207
E-mail: **dhilgenberg@fhi.org**

Irving Hoffman, PA, MPH
University of North Carolina
Center for Infectious Diseases
1700 Airport Road, CB # 3368
Chapel Hill, NC 27599 USA
Phone: 1-919-966-6324
Fax: 1-919-966-8536
E-mail: **Hoffmani@med.unc.edu**

Gift Kamanga, CO, MSc
University of North Carolina Project
Tidziwe Centre
Kamuzu Central Hospital
Private Bag A104
Lilongwe
Malawi
Phone: 011-265-1-755-056
Fax: 011-265-1-755-954
E-mail: **gkamanga@unclilongwe.org.mw**

Johnstone Kumwenda, MBChB, MSc, MRCP
College of Medicine-Johns Hopkins Project
P.O. Box 1131 Chipatala Avenue
Blantrye, Malawi
Phone: 265-884-1875
Fax: 265-167-0132
E-mail: **jonnykumwenda@yahoo.com**

Newton Kumwenda, M.P.H., Ph.D.
Field Director
Johns Hopkins Project
P.O. Box 1131
Blantyre, Malawi
Phone: 011-265-631-527
Fax: 011-265-631-527
E-mail: **jhopkins@sdnp.org.mw**

Diana Lynn
FHCRC-SCHARP
1100 Fairview Avenue N, LE-400
PO Box 19024
Seattle, WA 98109-1024 USA
Phone: 1-206-667-4980
Fax: 1-206-667-4812
E-mail: **dlynn@scharp.org**

Francis Martinson, MD, PhD
University of North Carolina Project
Private Bag A-104
Lilongwe
Malawi
Phone: 011-265-1-755-056
Fax: 011-265-1-755-954
E-mail: **fmartinson@malawi.net**

Catherine MacPhail, PhD
Reproductive Health and HIV Research Unit
Department of Obstetrics and Gynaecology
PO Box 18512
Hillbrow, 2038
South Africa
Phone: 011-27-83-441-5415
Fax: 011-27-86-504-1007
E-mail: **cmacphail@rhru.co.za**

William C. Miller MD, PhD
Associate Professor
Division of Infectious Diseases
2146 Bioinformatics
Campus Box 7030
University of North Carolina at Chapel Hill
Chapel Hill, NC 27599 USA
Phone: 1-919-966-9407
Fax: 1-919-966-2089
E-mail: **bill_miller@unc.edu**

Audrey Pettifor, PhD, MPH
University of North Carolina at Chapel Hill
2102-E Mcgavran-Greenberg
135 Dauer Drive
Campus Box 7435
Chapel Hill, NC 27599 USA
Phone: 1-919- 966-7439
Fax: 1-919-966-2089
E-mail: **apettif@email.unc.edu**

Kathleen Sikkema, PhD
Duke University
Department of Psychology and Neuroscience
PO Box 90086
Durham, NC 27708 USA
Phone: 1-919-684-9073
Fax: 1-919-660-5726
E-mail: **kathleen.sikkema@duke.edu**

Taha E. Taha. MD PhD
Johns Hopkins University
615 North Wolfe Street, E7138
Baltimore, MD 21205
Phone: 410-614-5255
Fax: 443-287-5188
Email: ttaha@jhsph.edu

David Vlahov, PhD
New York Academy of Medicine, Center for
Urban Epidemiologic Studies
1216 Fifth Avenue
Room 556
New York, NY 10029 USA
Phone: 1-212-822-7383
Fax: 1-212-876-6220
E-mail: **dvlahov@nyam.org**

Xuesong Yu, Ph.D
SCHARP-FHCRC
1100 Fairview Avenue N, M2-C200
PO Box 19024
Seattle, WA 98109, USA
Phone: 1-206-667-7008
Fax: 1-206- 667-4378
E-mail: **xyu@scharp.org**

HPTN 062

Feasibility and Acceptability Study of an Individual-Level Behavioral Intervention for Individuals with Acute and Early HIV Infection

PROTOCOL SUMMARY

Purpose: To evaluate the acceptability and feasibility of an enhanced, individual-level counseling intervention for individuals in the acute and early phase of HIV infection aimed at reducing risk behaviors.

Design: A multi-site, two-arm, randomized prospective study.

Study Population: Men and women 18 years of age or older diagnosed with acute HIV infection and enrolled in the Center for HIV/AIDS Vaccine Immunology (CHAVI) 001.

Study Size: A target group of 46 individuals with acute HIV infection.

Intervention: Participants will be randomized in a 1:1 ratio to one of two study arms as shown below:

Study Arm	Treatment
**Experimental Arm**<br><br>**n = 23 participants**	Standard HIV post-test counseling at enrollment (baseline), and standard risk-reduction counseling at follow-up<br>*plus*<br>Four enhanced counseling sessions between enrollment and week 2 and a fifth booster session at week 8
**Control Arm**<br><br>**n = 23 participants**	Standard HIV post-test counseling at enrollment (baseline) and standard risk-reduction counseling at follow-up

Duration and Follow-up: The study will be conducted over approximately 2.5 years. Participants will be enrolled for approximately 24 months. Participants will be followed weekly for the first 2 weeks and then monthly starting at week 4 for a total of 6 months from enrollment.

Primary Objectives:

1. To evaluate the feasibility of enrolling and delivering an enhanced individual-level counseling intervention to individuals in the acute and early phases of HIV infection

2. To evaluate the acceptability of an enhanced, individual-level counseling intervention among individuals in the acute and early phases of HIV infection.

Secondary Objectives:

1. To examine the effectiveness in reducing risk behaviors for HIV transmission of an enhanced, individual-level counseling intervention aimed at individuals in the acute and early phases of HIV infection.

2. To examine participant risk behaviors for HIV transmission and participant perceptions of infectiousness after the acute and early phases of HIV infection.

Primary Endpoints:

Feasibility:

- Proportion of participants in the experimental arm who receive the first four intervention sessions in the first 2 weeks after being informed of their diagnosis
- Proportion of participants in both arms who receive their respective counseling sessions between enrollment and week 8
- Proportion of participants in the experimental arm who receive the first four intervention sessions at any time point after enrollment

Acceptability:

- Measures related to intervention format, content, and perceived effectiveness

Secondary Endpoints:

Intervention effectiveness

Number of self-reported unprotected sex acts

Sexual behavior and perception

- Number of new sexual partners
- Number of concurrent sexual partners
- Participant perceptions of infectiousness

Sites:

Malawi and/or other non-US CHAVI clinical sites

1.0 INTRODUCTION

The HIV epidemic in sub-Saharan Africa is severe and has grown at alarming rates. A household survey of close to 12,000 15- to 24-year-old South Africans yielded estimates of HIV prevalence of 4.8 percent (95 percent CI 3.9-5.9) among men and 15.5 percent (95 percent CI 13.7-17.6) among women (Pettifor, Rees, et al., 2005). Surveys of the South African adult population (15- to 49-year-olds) have indicated HIV prevalence of 16.2 percent (Shisana, Simbayi, 2005). HIV prevalence in 2006 was 29.1 percent among antenatal clinic populations (South African Department of Health, 2007). According to the 2006 UNAIDS report, 14 percent of pregnant women seeking antenatal care in Lilongwe in 2001 were infected with HIV. Also in Lilongwe, in 2005 14 percent of males and females aged 15–49 were estimated to be living with HIV.

This protocol describes research to evaluate an HIV behavioral intervention for individuals diagnosed with acute HIV infection (AHI). Formative behavioral data collected from individuals with AHI in Africa have been used to translate current behavioral interventions for individuals living with established HIV to behavioral interventions for individuals in the acute and early phase of infection. This study will examine the feasibility and acceptability of the adapted individual-level behavioral intervention among individuals diagnosed with AHI in Africa.

1.1 Background

Individuals with acute or early HIV infection may be responsible for a substantial proportion of onward transmission of HIV infection, especially in sub-Saharan Africa. Among Ugandan discordant couples, transmission of HIV was at least 7 to 8 times more efficient among persons with early infection as it was among persons with established infection (Wawer, Gray, et al., 2005). A recent re-analysis of these data suggests that transmission could be up to 26 times more efficient during AHI (Hollingsworth, Anderson, et al., 2008). The results of early mathematical modeling studies supported the identification of early HIV infection as a critical driving force in the HIV epidemic (Koopman, Jacquez, et al., 1997). More recent models, however, have yielded variable estimates. Where HIV spread is in an early growth phase, early infection appears to be responsible for the vast majority of transmission (Kretschmar, Dietz, 1998; Jaquez, Koopman, et al., 1994). In contrast, in models of endemic HIV disease, the importance of AHI is highly variable, ranging from less than 1 percent to 82 percent (Xiridou, Geskus, et al., 2004; Pinkerton, 2007; Abu-Raddad, Longini, 2008; Fraser, Hollingsworth, et al., 2007). The extreme heterogeneity of the estimates is due to differences in population-specific assumptions and parameter values, "early HIV" definitions, and model structures. Recent modeling suggests that the role of AHI and early HIV infection is increased in the context of high sexual partner concurrency and/or very high rates of partner change. Given the short duration of AHI, limited transmission due to AHI can be expected if the index case is in a monogamous relationship. However, if the index case has concurrent sexual partners, the risk of onward transmission increases substantially. Multiple partners and concurrency are prevalent in sub-Saharan Africa (Halperin, Steiner, et al., 2004).

To prevent HIV infections more effectively, there is a need to understand better the factors that are driving rapid transmission. The high viral burden in both blood and genital secretions in the time following infection results in individuals with AHI being highly infectious (Pilcher, Tien, 2004; Pilchar, Shugars, 2001). Peak HIV viral load is seen in the first 3 to 4 weeks (at day 17 in blood and at day 31 in semen) followed by decay to nadirs and chronic levels by week 12 (Figure 1) (Pilcher, Joaki, 2007). In most contexts, individuals with AHI are unaware of their status and may believe themselves to be uninfected until testing with standard antibody assays reveals their positive status.

Figure 1 – Viral load levels during acute infection phase

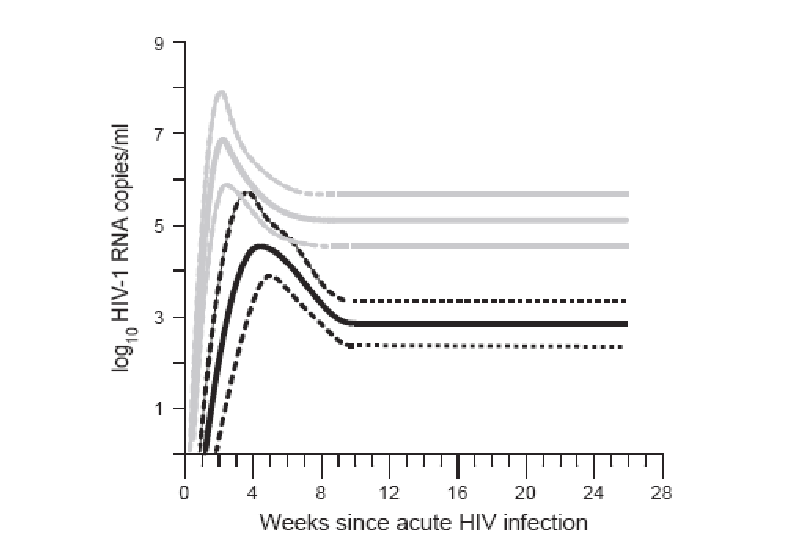

There are as yet no guidelines for behavioral prevention or biological management for individuals with AHI or their sex partners. With the technology currently available, identification of individuals with AHI is difficult and resource-intensive. In high-prevalence settings only approximately 1 percent of participants screened for AHI will test positive. However, technological advances in HIV testing in the future are likely to result in more widespread detection of AHI. In the meantime, there is an urgent need and a unique opportunity to intervene in this early stage when acutely infected individuals are highly infectious. Therefore prevention messages and best practices that clinicians and other health-care providers can promote with acutely infected individuals must be developed.

Specialized assays allow individuals with AHI to be identified and enrolled in programs to prevent the spread of infection (Stevens, Akers, et al.,2005; Pilcher, Fiscus, et al.,2005; Pilcher, McPherson, et al.,2002; Pilcher, Price et al.,2004). Health-care providers have three emergent obligations once they identify an individual with AHI:

- To consider sexual behaviors that put other people at risk for HIV and to intervene to address such behaviors. HPTN 062 addresses these issues.
- To consider the patient's sexual partners and sexual networks. Identifying sex partners is important to prevent further transmission of HIV, given that each individual with AHI has one or more partners who are already suffering from established infection (recognized or not), are themselves acutely infected (clusters of AHI have been common), or are HIV uninfected and could therefore benefit from counseling or other interventions. The importance of partners and partner referral/notification will be part of the intervention to be evaluated in HPTN 062.
- To decide whether the patient might benefit from antiretroviral therapy (ART), taking into consideration personal health and the possibility that some ART agents could rapidly reduce secondary transmission. Currently within government HIV treatment programs in Africa there is no precedent for immediate initiation of treatment for patients diagnosed early.

Until the AHI screening technology evolves, a phase-III trial of the efficacy of prevention intervention among individuals with AHI and their partners is not feasible. Therefore, prevention interventions must be developed using 1) existing empirical behavioral information about individuals with AHI and 2) information on effective prevention programs developed for individuals living with HIV. Prevention interventions must be tested for their acceptability and feasibility among individuals identified as acutely infected. Anecdotal reports from clinicians working with individuals with AHI suggest that individuals are eager to receive information at the time of diagnosis and that an intervention delivered at this time would be acceptable and feasible. Further, formative research from CHAVI 011 (see Section 1.2) indicates that individuals with AHI are interested in behavioral interventions during the acute phase of infection. Clinicians also believe there is good retention of prevention messages provided at this time (Hightow L, personal communication). There also appears to be a need to provide different or additional behavioral messages to individuals diagnosed with AHI compared to those with latent infection, given the immediacy of the risk behaviors contributing to the transmission of infection as well as the high level of infectiousness among those with AHI. These messages must also be carefully crafted and delivered to ensure that they do not in any way undermine the key risk-reduction messages applicable to persons with established HIV infection.

1.2 Preliminary Studies

Data are currently available from the CHAVI 001 and CHAVI 011 studies. These data describe the type of study population that would likely be enrolled in HPTN 062 and what participants want from a prevention intervention focused around the time of early infection.

CHAVI 001

CHAVI 001 is a prospective observational cohort study in which cross-sectional screening is performed to identify and enroll individuals with AHI in the US and Africa. Subjects with negative, indeterminate, or discordant antibody tests and detectable HIV ribonucleic acid (RNA) within 45 days of study entry who provide informed consent are eligible for enrollment. Specimens are collected for evaluation of cellular, antibody, and mucosal responses during AHI. CHAVI 001 provides the necessary infrastructure to engage individuals in HPTN 062 and also provides clinical care for all patients and psychosocial support and referral for those who need these services. Through CHAVI 001, a large number of subjects have been screened to identify and confirm about 1 percent with AHI, based on the algorithm being used in that study. To maximize the potential impact on transmission in HPTN 062, the time from detection (diagnosis) with AHI to initiation of the intervention should be as short as possible. As of August 2008, fewer than half of the participants were enrolled in CHAVI 001 within 2 weeks of diagnosis. The most timely enrollment has been in Lilongwe and Johannesburg (Table 1).

Table 1 - Time (days) from screening to enrollment in CHAVI 001			
**Site**	**N**	**Median (days)**	**Range (days)**
US	35	16	3-32
Lilongwe	16	7	1-13
Blantyre	9	19	7-44
Durban	19	28	11-42
Johannesburg	18	10	4-31

Recruitment of individuals with AHI from Johannesburg and Lilongwe (CHAVI 001): CHAVI 001 opened for screening and enrollment at sites in Johannesburg and Lilongwe in November 2006. As of August 2008, the site in Johannesburg screened approximately 2507 individuals and enrolled 23 in the study. The site in Lilongwe screened approximately 3423 individuals and enrolled 31 in the study. The overall prevalence of AHI among those screened to date at the sites in Johannesburg and Lilongwe is 1 percent for both sites. In the site in Johannesburg, 60 percent of the individuals with AHI are women and over 80 percent are age 25 years or younger. In Lilongwe, approximately 40 percent of individuals with AHI are women and 75 percent are 20 or younger.

HPTN 062 will be conducted ancillary to and parallel with CHAVI 001 and will recruit participants when they come for their initial CHAVI 001 visit. Enrollment in CHAVI 001 is currently planned to continue until 2011.

CHAVI 011

CHAVI 011 was a multi-method, qualitative, formative research study conducted in parallel with CHAVI 001 in Lilongwe, Malawi and Johannesburg, South Africa. The primary objective of CHAVI 011 was to facilitate implementation of CHAVI 001 by enhancing recruitment, retention, partner notification, and prevention counseling through the collection of formative research of individuals with AHI and their sex partners, and members of the community in South Africa and Malawi. While CHAVI 011 did not include the development or piloting of an intervention, a secondary objective was to collect behavioral data to inform design of a future behavioral intervention for individuals with AHI.

Data from CHAVI 011 that contributed to design of the HPTN 062 intervention include:

- Sexual behaviors of individuals during the period immediately surrounding acute HIV infection
- Characteristics of sexual partnerships among individuals during the period immediately surrounding acute HIV infection
- Changes in sexual behaviors since notification of diagnosis with AHI
- Types of interventions that individuals with AHI would need and want during the period immediately following notification of diagnosis with AHI, the best timing for the intervention, potential intervention content, and retention of messages provided at the time of notification of diagnosis with AHI

CHAVI 011 provided an excellent opportunity to understand the behaviors of individuals with AHI and the factors important to address in an intervention designed specifically for these individuals. A summary of preliminary data from CHAVI 011 follows.

Summary of findings from CHAVI 011

Through CHAVI 011 key behaviors that must be addressed as part of a behavioral intervention were identified. In-depth interviews with 37 participants with AHI in Johannesburg (n=18) and Lilongwe (n=19) were conducted from October 2007 to June 2008. Interviews were conducted at one week, one month, and three months post-diagnosis with AHI for participants who were newly enrolled in CHAVI 001 after the start of CHAVI 011 (n=19). Participants who were already enrolled in CHAVI 001 when this study started took part in only one interview (n=18). Interviews were conducted in the local language of choice, audio taped, and then transcribed and translated. Data were then coded in Atlas-TI or Nvivo. Next, code reports were generated and matrices developed for the major themes and patterns arising from the data related to sexual behavior.

Key findings from CHAVI 011 include the following:

Sexual behaviors: Most individuals with AHI in South Africa and many individuals with AHI in Malawi reported continuing to have sex post-diagnosis. Many participants also reported changing their sexual behaviors after diagnosis. Changes included abstaining from sex, reducing the number of partners, drinking alcohol less often, and/or using condoms consistently or more often. Major motivators for safer sexual behavior were fear of re-infection and fear that unsafe sex would lead to a decline in health. In both countries, about half of the participants reported using condoms and half reported inconsistent or non-use. Condom use appeared to be related to concerns about re-infection, knowledge of a partner's HIV status, and type of partner (i.e. casual or main partner).

Issues related to individual's understanding of AHI: The majority of participants with AHI in Malawi and many participants with AHI in South Africa were unaware that they were more infectious during the acute phase of infection. However, most participants understood that AHI is a recent infection. Common misperceptions about AHI included:

- The infectiousness of individuals with AHI and individuals with established HIV infection are the same.
- Individuals with established infection are more infectious because they have had the virus longer and therefore have more viruses.
- Individuals with AHI are more infectious because they do not know they are infected and therefore do not practice risk reduction methods.
- Individuals with AHI are less infectious as there are no obvious signs and symptoms of the illness compared to when individuals have AIDS.

Issues related to potential interventions for individuals with AHI: All participants in South Africa and almost all participants in Malawi reported that they would be interested in an intervention offered at the time of diagnosis that would help with safer sex behaviors and coping with the diagnosis. Intervention topics of interest that were mentioned commonly by participants included:

- Using condoms
- Disclosure self-efficacy skills
- Concerns about infecting others
- Partner violence/ being safe in a relationship
- Ways to be intimate with one's partner
- Alcohol and drug use
- Talking to one's children about being HIV positive
- Having sex with someone who is HIV positive

Additional site-specific information related to potential interventions – Malawi:

The ideal schedule for a series of intervention sessions ranged from once a week to once a month, and the time most commonly preferred for the intervention sessions to begin ranged from the day of diagnosis through one week post-diagnosis. Participants with AHI indicated that abstinence after diagnosis might be possible with appropriate counseling. However, participants acknowledged that both desire for sex and non-disclosure to partners will be barriers. Participants with AHI also indicated that a 100-percent rate of condom use post-diagnosis might be possible. Barriers they identified included the need to disclose and the difficulty in using condoms in steady relationships in which condoms had not been used previously. The following potential topics for intervention were also commonly mentioned:

- Abstaining from sex
- How to be safer during sex
- Worries about getting a sexually transmitted infection
- Future relationships
- Feeling afraid of one's partner

Additional site-specific information related to potential interventions – South Africa: Participants mentioned the importance of having a place to talk about their diagnosis and having supportive people who can listen to them, particularly in a climate where stigma surrounding HIV can make many people feel isolated and alone. Most participants reported that they do not think it is possible to abstain from sex for three months after diagnosis with AHI. The majority of participants did think it would be possible to use condoms consistently for a three-month period during AHI. However, a number of barriers to condom use were reported – for example, lack of motivation to use condoms, male partners refusing to use condoms, lack of knowledge about how to use condoms correctly, lack of trust in condoms, and the desire to have children. The following potential topics for intervention were also commonly mentioned:

- Talking with ones partner about sex
- Nutrition and healthy living
- Having a baby when HIV positive

Issues related to disclosure of HIV status: The vast majority of participants with AHI disclosed their status to someone -- a sexual partner, family member, or friend -- in the period after diagnosis. Participants were concerned about rejection, gossip, and rumors as a consequence of disclosure. They said that interventions could help people with AHI determine the best way to disclose their status to their families and partners.

Issues related to partner notification: Extensive data were collected on the advantages, disadvantages, preferences, and perceived partner reactions to partner notification approaches from individuals with acute HIV infection, partners of individuals with established HIV infection, research staff, community leaders, and individuals with established HIV infection.
Finding included that participants said that people should be informed of their partners' HIV status. Advantages and disadvantages of both approaches were identified. In Malawi, although support for patient notification did not strongly outweigh support for provider notification among the participant groups, more individuals with AHI in Malawi preferred patient notification. In South Africa, individuals with AHI preferred patient and provider notification equally. In both sites, participants suggested couples counseling as another approach to partner notification where partners be tested together, even if one partner has already learned his/her status. Participants in both sites also commonly expressed the fear that partners could respond negatively and that they could experience stigma as a result of partner notification. In Malawi, while violent reactions to disclosure were not reported during the interviews with participants with AHI, disclosure often led to the termination of relationships. Among this group of participants with AHI, several female partners chose to leave their male partner after he was diagnosed with acute HIV

infection. Overall, fears of partner's reactions and fears of discrimination from family/friends/community hindered disclosure (as did the perceived limited opportunities to disclose one's status). In South Africa, participants with AHI were also concerned about rejection, gossip and rumors as a consequence of disclosure. A single participant experienced a social harm event related to her partner disclosing her status to others.

Given diverse perspectives and individual situations, a combination of approaches to partner notification may be needed; offering the option of provider-assisted notification may be helpful.

In summary, the CHAVI 011 data suggest that participants with AHI would accept a counseling intervention focused specifically on changing behavior during the acute phase of HIV infection to avoid transmission of HIV to uninfected partners. As presented above, participants identified several topics that should be included in such a counseling intervention and these are integrated into the intervention developed for HPTN 062.

1.3 Study Rationale

Individuals with acute or early HIV infection may be responsible for a substantial proportion of onward transmission of HIV infection, especially in sub-Saharan Africa. Several recent modeling studies have addressed this issue with varying results (Hollingsworth, Anderson, et al.., 2008; Pinkerton 2008; Abu-Raddad, Longini; 2008). However, such individuals are widely believed to be more than 20 times more infectious than those with established/chronic HIV. Researchers generally believe that transmission from individuals with AHI accounts for a substantial share of new cases, depending on a number of factors such as stage of the epidemic and partnership patterns.

Existing technology makes it difficult to identify large numbers of individuals with AHI. However, future technological advances in HIV testing will make it easier to identify such individuals earlier and in larger numbers. Currently, rapid HIV tests are being developed to identify individuals very soon after infection. As these new technologies become available, data on behavioral interventions designed specifically for individuals in the acute phase of infection will be needed. HPTN 062 will pilot and evaluate an intervention designed specifically for this purpose. Given the substantial infrastructure required to identify individuals with AHI, HPTN 062 will be implemented ancillary to and in parallel with CHAVI 001, an acute HIV-1 infection prospective cohort study in sub-Saharan Africa.

The HPTN 062 intervention is different from the standard-of-care counseling being provided as part of CHAVI 001. While many of the topics provided in the HPTN 062 intervention are the same as those covered in CHAVI 001, the approach used to discuss these topics is quite different (e.g., motivational interviewing). HPTN 062 also includes a wider array of topics than is covered in standard CHAVI 001 counseling. Role playing, skills building, and other activities will be implemented to foster self-efficacy and to increase motivation to practice safer sex. These approaches/techniques are not part of standard counseling offered through CHAVI 001. Based on the information available from CHAVI 001, no counseling scripts exist with specific messages for AHI. Therefore, the enhanced HPTN 062 intervention is different from the standard counseling offered through CHAVI 001 in the following ways: It is systematic, theory-based, aims to enhance skill-building, provides more in-depth information, includes development of a patient-focused risk-reduction plan, and allows more time for the key topics to be covered.

The proposed intervention is different from existing "positive prevention" programs in that no current interventions focus specifically on the acute or early phase of HIV infection. While components of the HPTN 062 intervention are similar to other skill-building approaches being

used in positive-prevention programs, the HPTN 062 intervention incorporates empirical data on sexual behavior among individuals with AHI, including desires for interventions and understanding of AHI. Thus HPTN 062 focuses on issues identified as most salient to these individuals. In addition, the timeframe in which the intervention is being delivered is a key feature distinguishing it from existing positive-prevention programs. Specifically, the HPTN 062 intervention starts on the day that participants are informed of their AHI diagnosis, at a time when they are highly infectious and thus concentrating primarily on reducing risk during a targeted versus indefinite period. For individuals diagnosed with acute or early HIV infection through studies or screening programs, there is an ethical imperative to provide guidance for behavior change to limit transmission to uninfected partners, particularly during this highly infectious phase.

2.0 STUDY OBJECTIVES AND DESIGN

2.1 Primary Objectives

1. To evaluate the feasibility of enrolling and delivering an enhanced individual-level counseling intervention to individuals in the acute and early phases of HIV infection.

2. To evaluate the acceptability of an enhanced, individual-level counseling intervention among individuals in the acute and early phases of HIV infection

2.2 Secondary Objectives

1. To examine the effectiveness in reducing risk behaviors for HIV transmission of an enhanced, individual-level counseling intervention aimed at individuals in the acute and early phases of HIV infection.

2. To examine participant risk behaviors for HIV transmission and participant perceptions of infectiousness after the acute and early phases of HIV infection

2.3 Study Design

This is a multi-site, two-arm, randomized, prospective study to evaluate the feasibility and acceptability of a counselor-delivered behavioral intervention for individuals identified as having AHI or early HIV infection.

Individuals in the acute/early phase of HIV infection who are participating in CHAVI 001 will be recruited for HPTN 062. For the purposes of this study, the following definition of AHI from CHAVI 001 will be used:

Documentation of the following within 45 days of enrollment:

- Concordant negative or discordant HIV antibody tests using two different rapid HIV tests, OR a negative HIV EIA test

  AND

- Positive nucleic acid test (NAT)

After being informed of their diagnosis of AHI, participants in both study arms will receive the same standard post-test HIV counseling aimed at risk reduction in conjunction with HIV testing through CHAVI 001. Individuals enrolled in CHAVI 001 who are interested in participating in

HPTN 062 will go through the process of informed consent for HPTN 062. Participants will then be randomized in a 1:1 ratio to one of two study arms. All participants will be followed for 6 months post-enrollment. Participants in both arms will receive standard HIV risk-reduction counseling at weeks 1-4, 8, 12, 16, 20 and 24 through CHAVI 001. (This is in accordance with standard CHAVI 001 procedures.) See section 4.2 for a description of standard risk-reduction counseling

Participants randomized to the experimental arm will take part in four enhanced individual counseling sessions (from enrollment to week 2) which aim to help reduce the risk of sexual transmission of HIV to their uninfected partners, particularly during this highly infectious initial stage. The introductory session will be delivered after the participant has been informed of his/her diagnosis, has received post-test counseling through CHAVI 001, and has provided informed consent for HPTN 062. Three subsequent sessions (sessions 2, 3 and 4) will be delivered between the introductory session and week 2. The fifth session -- a booster and the final session of the series -- will be provided at week 8. This session will focus on maintenance of risk-reduction behaviors during the established phase of HIV infection. See Section 4.1 for a description of the experimental intervention sessions. During the last 4 months, participants in both arms will be followed monthly to collect information on behavior change and perceptions post-intervention. HIV risk behaviors and perceptions will be compared across the two arms of the study. The feasibility and acceptability of the experimental intervention will be evaluated.

Note: While the burden on individuals who have recently been diagnosed with HIV must be limited, this concern must be balanced with the need to provide prevention messages for individuals with AHI as soon after diagnosis as possible to maximize the benefit in reducing HIV transmission. Formative data from CHAVI 011 suggests that individuals want to talk with someone about their diagnosis; individuals reported wanting an intervention during the acute phase of infection.

Note: Acknowledging the small sample size of the study, inclusion of a randomized comparison offers a number of advantages over a non-randomized, non-controlled study design. Without a comparison group, there could be no assessment of whether any differences in behavior observed over time may have resulted from the intervention. Another advantage is that it will allow for assessment of an effect size, which will be valuable if a larger study is to be conducted. Non-randomized, non-controlled trials are also more susceptible to bias and confounding.

All participants will be asked a behavioral assessment at enrollment (baseline) and at weeks 1, 2, 4, 8, 12, 16, 20 and 24. The behavioral assessment will be conducted prior to the CHAVI counseling sessions, except at baseline, when the assessment will be conducted after the CHAVI post-test counseling. Topics that may be covered are listed in Table 4. A brief post-session quantitative assessment will be administered to participants after each experimental intervention session (for the experimental group) and after each standard-of-care counseling session at enrollment and at weeks 1 and 2 (for the control group) to collect information about the acceptability of each individual session (i.e., content, format, and perceived effectiveness) and to document topics discussed. A separate post-session questionnaire will also be administered to each arm at different assessment time points and will cover HIV knowledge, risk perceptions and self-efficacy.

An in-depth interview (e.g. qualitative semi-structured interview) will be conducted with participants at weeks 2, 8, 12, and 24, in both arms. At the week 2 interview, questions will primarily focus on participants' perceptions on the format, content, and effectiveness of either the experimental intervention sessions or the standard-of care counseling sessions, depending on their

assigned study arm, in order collect more descriptive information on the acceptability of the intervention. For participants in the experimental arm, they will also be asked to describe whether or not they perceive any further benefit from attending the experimental intervention sessions in addition to the standard-of-care counseling they received. Questions on sexual behavior will be asked as appropriate to enhance the quantitative data collected on intervention effectiveness and on sexual behavior and perception. At the week 8, 12, and 24 interviews, questions will primarily focus on gathering more in-depth information on sexual behavior and on perceptions of risk behavior with an emphasis on investigating changes in participants' risk-reduction behavior as they transition from the acute/early phase of infection into established infection. All qualitative interviews will be audio taped if permission is granted by the participant.

The behavioral assessment will be administered prior to counseling (except at baseline) by staff other than the counselor, in order to reduce a patient's inclination to provide socially desirable responses to questions about his/her sexual behavior.

Note: Information on social harms will be collected as part of CHAVI 001. In addition, social harms judged to be significant by the Investigator of Record will be reported to the relevant IRB/ECs at least annually, or according to their individual requirements.

Counselor Interviews: It is anticipated that one counselor will deliver the experimental intervention at each site; however, sites may choose to have more than one counselor deliver the intervention. Written informed consent will be obtained from the counselor(s) who deliver the experimental intervention (see Appendix II B for a sample study consent document for counselors) prior to participation in the interview. Approximately three qualitative interviews will be conducted with each counselor(s) at each site to capture their perceptions of the acceptability and perceived impact of the intervention over the course of the study. Topics that will be explored include: perceived effectiveness of the intervention, strengths and weaknesses of the intervention, areas for improvement, challenges of delivering the intervention, challenges of following the experimental intervention curriculum, perceptions of participants' experiences, and other topics related either to the content, format, perceived effectiveness of the intervention, or perception of the participants' experiences.

Both the counselors who deliver the experimental intervention and the counselors/clinicians who provide standard counseling as part of CHAVI 001 will complete a brief quantitative assessment at the end of each session to document topics covered in each session and the goals set. This assessment will take place after every intervention session in the experimental arm, and at baseline, week 1 and 2 in the control arm. Counselors will also provide a summary of the session discussions, including strategies discussed.

3.0 STUDY POPULATION

This study targets enrollment of approximately 46 men and women 18 years of age or older diagnosed with AHI during the enrollment period of approximately 24 months.

Note: A minimum of 46 participants is targeted; however, the final number may be higher or lower, depending on the rate of accrual in CHAVI 001 during the HPTN 062 enrollment period of approximately 24 months.

3.1 Inclusion Criteria

To be eligible for inclusion in the study individuals must:

- Be enrolled in CHAVI 001

Note: The inclusion and exclusion criteria for CHAVI 001 are provided below. The criteria will not be reassessed prior to enrolment in HPTN 062.

Inclusion Criteria from CHAVI 001

- Men and women aged ≥ 18 years at the time of screening.
- Able and willing to provide adequate information for locator purposes.
- Hemoglobin > 10.0 g/dL.
- Willing to receive HIV test results.
- Not intending to relocate out of the area for the duration of study participation and does not have a job or other obligations that may require long absences from the area.
- Has acute HIV infection. (See Section 2.3 for definition).

Exclusion Criteria from CHAVI 001

- Any prior or current use of antiretroviral therapy (ART). This does not apply to individuals screening for Group 1A. ART use for the prevention of perinatal transmission may be waived after prior consultation with the protocol team.
- Previous or current participation in a HIV vaccine study.
- Any condition that, in the opinion of the Investigator of Record, would make participation in the study unsafe, complicate interpretation of study outcome data, or otherwise interfere with achieving the study objectives.

Note: Counselors at each site who deliver the experimental intervention and provide informed consent will also be interviewed. A sample of the consent form is in Appendix IIB.

3.2 Recruitment Process

Individuals in the acute phase of HIV infection will be recruited from CHAVI 001. Once identified as having AHI by CHAVI 001, the individual will be introduced to the HPTN 062 study. This will occur after they are informed of their AHI diagnosis, receive standard HIV post-test counseling as part of CHAVI 001, and are fully enrolled in CHAVI 001. Individuals interested in participating in HPTN 062 will be asked to provide written informed consent for participation after the study has been fully explained to them. A sample of the consent form is in Appendix IIA. It is anticipated that approximately 1-2 individuals with AHI will be enrolled per month at each site.

3.3 Participant Randomization

Participants will be enrolled and randomized after providing informed consent and determination of eligibility. Participants will then be randomized in a 1:1 ratio to either the experimental arm or the control arm. Study staff will inform individuals of their randomization assignment at the enrollment visit.

3.4 Participant Retention

Enrolled participants will be followed for 6 months. The retention rates for this study will benefit by being implemented together with CHAVI 001, which actively follows up with all participants. For example, study sites will attempt to schedule study visits and intervention sessions when participants are already scheduled to come to the clinic and will use the locator information and tracing procedures already in place for CHAVI 001.

3.5 Participant Withdrawal

Participants may withdraw from the study at any time. The Investigator of Record also may withdraw participants from the study in order to protect their safety and/or if they are unwilling or unable to comply with required study procedures after consultation with the Protocol Chair, Division of AIDS (DAIDS) Medical Officer, HPTN Statistical and Data Management Center (SDMC) Protocol Statistician, and Coordinating and HPTN Coordinating and Operations Center (CORE) Protocol Specialist.

Participants also may be withdrawn if the study sponsor, government or regulatory authorities, or site institutional review boards/ethics committees (IRBs/ECs) terminate the study prior to its planned end date.

Every reasonable effort will be made to complete a final assessment of participants who leave the study prior to the planned termination date and study staff will record the reason(s) for all withdrawals from the study in participants' study records.

Participants will be informed that withdrawal from the study will not affect their continued receipt of HIV services at the clinic or participation in CHAVI 001 in any way.

4.0 BEHAVIORAL INTERVENTIONS

4.1 Experimental Enhanced Individual Counseling

The intervention used in this protocol draws on empirical behavioral research findings on individuals with AHI with regard to sexual behaviors and perceptions of possible prevention interventions for individuals with AHI. This information has been combined with effective interventions for individuals living with HIV/AIDS that have been evaluated or are currently being evaluated.

While there are no behavioral interventions for individuals with AHI, a number of interventions have been evaluated for individuals living with HIV in the US, and some of these are currently being implemented in African settings. The characteristics of interventions that have been found to be effective in reducing unprotected sex among individuals living with HIV/AIDS were taken into account when developing the HPTN 062 intervention.

Given evolution of the viral set point over a few weeks, the behavioral intervention needs to be delivered intensively and early after diagnosis with AHI if it is to have an immediate impact on transmission. The key elements of existing positive-prevention interventions and best practices with regard to AHI were incorporated meaningfully into the intervention. The intervention will be delivered on an individual basis (not group) due to the manner in which individuals with AHI are identified and due to the time-related urgency of delivering the intervention as soon after diagnosis as possible.

Individuals randomized to the experimental arm will receive an introductory intervention session and will then receive three intervention sessions (Sessions 2, 3 and 4), and a final booster session (Session 5) -- all described in detail below. The introductory intervention session will be delivered after the participant is informed of his/her diagnosis and has received standard post-test counseling. Ideally this will occur on the day of enrollment but time constraints may defer it. Three subsequent sessions will then occur between the introductory session and week 2. The fifth, booster session will be delivered two months after enrollment. Although this intervention will focus on individuals with AHI/early infection, a component of the intervention will be to encourage the notification of sex partners. Each intervention session will be approximately one hour long, except the introductory session, which will be shorter.

4.1.1 Theoretical Underpinnings

A characteristic that successful behavior-change interventions for individuals infected with HIV share is that they are tailored to behavioral theory (Colfax, Buchbinder, et al., 2002). The intervention proposed is based on the Information, Motivation, Behavioral Skills (IMB) model. The IMB model assumes that information and motivation trigger behavioral skills that are required to change behavior. Another rationale for using IMB is that a recent meta-analysis of randomized, controlled trials of HIV secondary prevention interventions found that successful interventions contained motivational and skill-building elements (Crepaz, Lyles, et al., 2006). While the intervention used in this study will be guided by behavioral theory, it will also be innovative, with a multi-disciplinary approach that ensures the greatest chance of being successful in changing behaviors.

4.1.2 Experimental Intervention Content

HPTN 062 draws from the content of interventions developed to reduce risky sexual behavior for people living with HIV/AIDS, including the Options Project (Fisher JD, Fisher WA et al., 2006), and Project Safe Talk (Golin, Patel, et al., 2007). It also uses empirical findings from CHAVI 011, which are summarized in Table 2. All of the positive-prevention interventions examined are based on the IMB model, using motivational interviewing as a guide. An important element of the proposed intervention is that it is patient-centered. Intervention sessions are tailored to each participant's needs. Counselors help participants to identify their own reasons to change and strategies to achieve change. Participants work to improve decision making skills and identify an appropriate course of action. For example, participants who have not yet begun to consider changing their sexual behavior will focus on different issues and goals than those who periodically or routinely practice safer behavior. For patients who report no risk behavior, the focus of the intervention is on reinforcing and maintaining their safer behavior and preventing lapse or relapse into risky behavior.

Given that AHI is brief, to effect behavior change the intervention will balance directive messaging given to all participants with counseling tailored to the participants' particular circumstances. The directive messages that counselors will deliver at each session will cover the following topics: 1) understanding AHI and HIV infection, 2) abstinence and condom use, and 3) partner notification and disclosure. Once key informational elements of the intervention are covered, the participants will be able to choose topics relevant to their particular situations. In keeping with the IMB and motivational interviewing framework, a counselor will assess a participant's current knowledge at each session and provide new information, as needed; assess motivation for behavior change; and assess self-efficacy to practice safer sex (behavioral skills). The session will be tailored to the area(s) needing most attention (Steps 2-5, Table 3). At the end

of each session, participants will set a behavioral change goal and receive a prescription for this goal (Step 7, Table 3).

Note: The initial sessions of prevention interventions designed for individuals with *established* HIV infection typically focus on building rapport between the counselor and the patient rather than discussing specific risk-reduction behaviors. Given the time-sensitive nature of AHI, counselors delivering this intervention still must focus on establishing rapport but will also assess the participants' readiness for new knowledge and provide new information using the Elicit-Provide-Elicit technique used in motivational interviewing. If during any session an individual mentions suicidal ideation or extreme depression, the counselor will ensure that appropriate measures are taken to follow-up with trained professionals with clinical expertise in this area.

Table 2 - Link between intervention session content and empirical data (CHAVI 011)

**Intervention Component (Session Number)**	**Link from Empirical Research**
Understanding AHI (Sessions 1 and 2 and follow-up at each session)	Limited understanding of AHI
Abstinence and condom use (Sessions 1 and 2 and follow-up at each session)	Participants continued to have sex post-diagnosis; condom use inconsistent
Partner notification (Sessions 2 and 3 and follow-up at each session)	Participants indicated that people should be informed of their partners' HIV status. Advantages and disadvantages of both patient- and provider-notification were identified; more participants preferred patient notification but acknowledged that provider notification is appropriate in some situations.
Condom-use skills and negotiation (Session 3)	Condom use is inconsistent. Even in long term, primary partnerships condom use is difficult. Correct condom-use skills appear needed.
Disclosure and social support (Session 3)	Most participants have disclosed to someone (family, friend, or partner) in the time following diagnosis but need disclosure, self-efficacy, and decision making skills.
Topic choice (Session 4)	Participants raise many other topics of interest -- e.g., having a baby when positive, alcohol and drug use, partner violence, being intimate with partners.
Maintenance (Booster)	Participants seem confident that they can practice safer sex for a defined period (i.e., 3 months). However, ensuring that participants have a longer-term plan post-intervention and AHI-period is important. Also important is ensuring that participants are linked to care and are aware of their long-term prevention and care options and strategies.

Table 3 - Counselor activities during each behavioral intervention session

Step	Counselor Activity
1	Explicitly assess the participant's current relationship status, sexual activities, and risk behaviors; identify current HIV-transmission risk behavior and conditions under which the risk behavior may occur. If multiple risk behaviors are identified, the participant will choose one to focus on during the current visit.
2	Assess the participant's readiness to change behavior or maintain safer sex -- specifically his/her motivation, including responsibility and self-efficacy (confidence) to address the chosen behavior.
3	Tailor the session to fit the participant's individually assessed needs, situations, and behaviors, including barriers to enacting the behaviors and strategies to overcome those barriers.
4	Respond to a range of participant situations using a menu of standardized counseling strategies.
5	Deliver standardized informational messages, using the Elicit-Provide-Elicit technique of Motivational Interviewing.
6	Negotiate a goal or plan of action with the participant.
7	Document and "prescribe" the negotiated goal or action plan for the participant. The prescription component is derived from the Options intervention and is consistent with cultural norms in sub-Saharan Africa that place value on medical advice and prescriptions.

The overall outline for the intervention is described below. Some variation may occur during the development of the study manual and counseling guidelines.

Session 1: Participants should receive this introductory session on the day they receive their HIV diagnosis. In this session, the counselor will introduce to the participant the aims of the intervention and review the diagnosis of HIV infection. The primary goals of the first session are to 1) establish rapport between the counselor and participant, 2) allow the individual to begin to process the diagnosis, 3) begin the process of ensuring that the participant understands what acute HIV infection is and the greater degree of transmissibility that exists during AHI, 4) encourage abstinence or 100-percent condom usage if the participant chooses to have sex, and 5) partner notification, if the participant is comfortable with disclosure. The counselor will emphasize developing a risk-reduction strategy focusing on safer sex practices for the upcoming week.

At the end of this session ***and all subsequent sessions***, the individual will set a behavior-change goal related to safer sex and will receive a "prescription" related to this goal.

Session 2: This ***and all subsequent sessions*** will begin with a review of the participant's emotional status, understanding of HIV, behavioral experiences relating to sex or abstinence, and barriers to the chosen behavioral goals. The counselor will also review the status of the process of partner disclosure/notification and referral. Given the stress of receiving a diagnosis of HIV, the participant may not have absorbed much of the information provided in the first session. Therefore, the counselor will provide greater detail regarding the main topics described below, assess the participant's understanding of these topics, and assess the participant's readiness to learn more.

Understanding AHI: The counselor will emphasize the increased infectiousness during this period and the high probability of transmitting the virus to a partner. An important goal of this session is to ensure that the participant understands the meaning of the diagnosis, including the diagnosis of AHI.

Abstinence and Condom Use: The counselor will discuss the hierarchy of HIV risk and ensure that the participant understands modes of transmission. The counselor will explain that abstinence is the only method that is 100-percent effective in preventing sexual transmission of HIV, but that if a person does have intercourse, 100-percent condom use is the only other option. The concept of re-infection will be introduced. The participant's motivation and self-efficacy to abstain from sex or to practice safer sex will be assessed. The counselor will introduce the notion of responsibility to one's partners and community as part of the motivation to practice safer sex. Participants who say they are highly motivated to use condoms but who doubt their competence to use them correctly will have their session tailored to provide more focus on building their skills to use condoms. Participants with low motivation may need more discussion about why it is important to use condoms than information on how to use them.

Partner Disclosure/Notification and Referral: The counselor will discuss the importance of notifying people with whom the participants have had sex within the past 12 weeks that they have had sex with someone who has been diagnosed with HIV and the importance of referring them for HIV counseling and testing. Counselors will describe to participants the two main methods for notifying and referring partners – *patient* notification and referral (where the participant informs and refers her/his sex partners) and *provider* notification and referral (where a provider informs and refers the participant's sex partners). Participants who wish to notify their sex partners themselves can choose to do so in the way that is most comfortable for them. The counselor will explore with the participant her/his perceptions of the partner's response, and assist as needed in order to limit any negative consequences that could arise from disclosure. The counselor will follow up on this issue at each session in order to encourage partner notification but also to ensure the safety of the participant. If requested, the counselor will visit the participant's sex partner in her/his home, or at another location, in order to notify the sex partner of the participant's status. Depending on the wishes of the participant, she/he may or may not be present when the counselor speaks with the sex partner. Couple counseling will be provided as another means in which to inform partners. Emphasis will be placed on disclosure to steady and other current sex partners, because data from CHAVI 011 demonstrate that disclosure is necessary for participants to be able to practice safer sex behaviors.

Counselors will also discuss the concept of contract notification. A participant who agrees to this will have 1 or 2 weeks to disclose her/his status to a partner. Thereafter a counselor will disclose to the partner (with the consent of the individual). For those who do not agree to contract notification, the importance of partner notification will be discussed nonetheless, as described above. Given the high proportion of sero-discordant couples in Africa, counselors will introduce the concept of sero-discordancy and discuss in a supportive, nonjudgmental manner the pros and cons of partner notification and disclosure at this time. An important element of this topic is the benefit to participants of identifying sources of support and coping mechanisms to manage their diagnosis.

Counselors will also discuss the reasons why participants should disclose their status to any people with whom they plan to have sex in the future.

Session 3: In addition to reviewing the key elements from the previous session, Session 3 will focus on learning condom-use skills and negotiation, disclosure-communication skills, and social support.

Condom-use skills and negotiation*:* In this session the counselor will teach skills for the correct use of condoms and, using role play, techniques for negotiating safer sex.

Disclosure and social support: Building on the disclosure discussion which began during the first session, we will discuss the importance of partner notification and of disclosure and referral. In this session, we will focus on increasing decision making skills surrounding disclosure decisions and identify personal and environmental factors related to disclosure decisions. An important and related element of disclosure is social support. In this session participants will learn to identify types of social support (emotional, informational, and material) and identify positive and negative sources of social support. Participants will identify their personal social support networks relevant to each supportive category. Participants will identify personal stressors and determine which support categories would be most appropriate for each identified stressor. They will then identify who in their social network would be most helpful in providing support for each stressor. Disclosure will be discussed throughout the intervention.

Session 4: Session 4 will start by reviewing the participant's emotional status, HIV understanding, behavioral experiences relating to sex or abstinence and barriers to the chosen behavioral goals, and the status of partner notification/disclosure and referral. The focus of Session 4 will be discussion of a topic tailored to the participant's needs.

Topic Choice: In this session, individuals will be given a choice of possible topics to discuss, including: alcohol and drug use; fertility desires and options; ways to be intimate; feeling safe in relationships; feeling afraid of one's partner; maintaining abstinence; condom use; having sex with someone who is HIV positive; telling someone (including one's children), "I am positive"; how to be safer during sex; concerns about infecting others; worries about getting a sexually transmitted infection; future relationships; and nutrition and healthy living. These are topics that have been mentioned as important in our preliminary research on interventions during AHI. Participants will be able to choose one topic and then will follow the same intervention flow in terms of discussing its relevance.

Booster Session: The booster session will begin by assessing the emotional status of the participant. The session will continue with a review of each of the three directive messages (i.e., acute infection, safer sex, and disclosure) of the intervention and the participant's status with each component. The counselor will also emphasize maintaining healthy behaviors and explore risk disinhibition, given that the participant may no longer be considered in the highly infectious phase.

Maintenance: A major goal will be to maintain safe sexual behavior, specifically with regard to onward transmission. In addition, emphasis will be given to encouraging personal health, including engaging in the health-care system, understanding the importance of monitoring one's CD4 count, and preparing for ART use in the future. The participant will be assisted with planning for the future, including developing a support system. This will involve discussing the participant's social network and social support and identifying resources for maintaining support.

Risk disinhibition: After the acute phase is over, some participants may feel that the risk of transmission is much lower and therefore practice fewer risk-reduction behaviors. Counselors will continue to stress that transmission can also occur after the acute phase and discuss how to maintain safe sexual behavior.

4.1.3 Experimental Counselor Selection and Training

In each site, it is anticipated that one designated counselor will be selected to deliver the experimental intervention. Sites may choose to have more than one counselor deliver the intervention; however, counselors cannot deliver both the experimental intervention and standard-of-care counseling to avoid content contamination of the intervention. The counselor chosen to deliver the study should have experience conducting HIV counseling and will be provided study-specific intervention training. An important part of the training will include Client Centered Counseling and Motivational Interviewing techniques. In addition, he/she will be trained using the Safe Talk and Options safer sex training manuals that have been adapted for use with this intervention. The training will also cover the protocol in detail. A combination of didactic, interactive, and video sessions may be used in the training, and these approaches will teach the counselors to: 1) build and practice particular motivational interviewing counseling skills; 2) acquire and build sexual risk assessment skills; 3) increase knowledge of HIV infection (specifically AHI), safer sex methods, human sexuality, sexual health, a range of sexual behaviors, as well as barriers to and facilitators of safer sex practices; 4) become adept at following the steps of the intervention guide; and 5) integrate knowledge of safer sex into the context of intervention sessions.

To prevent contamination between study arms, counselors who receive the experimental intervention training and who provide the experimental intervention will not interact with control subjects and will not deliver standard risk-reduction counseling.

4.2 Standard Risk Reduction Counseling

All participants as part of CHAVI 001 (in which they will be co-enrolled) will be provided risk reduction in the context of post-test counseling at enrollment (baseline) and risk-reduction and safe-sex counseling sessions at weeks 1-4, 8, 12, 16, and 24. The risk-reduction and safe-sex counseling may also be administered to couples and is provided in accordance with local standards and regulations. The HPTN 062 protocol team will work with the CHAVI 001 counselors and clinical staff to ensure that the risk-reduction counseling provided through CHAVI 001 is consistent and “standard” across sites, while adhering to local guidelines. In addition counselors will be asked to indicate topics discussed at the end of each counseling session in order to compare topics discussed to the experimental intervention. Each session will typically consist of positive living counseling, which includes good nutrition, psychosocial support, and safe-sex counseling to prevent transmission to uninfected partners as well as re-infection.

4.3 Standardization and Quality Control

Ideally, an experimental behavioral intervention would be standardized. Although the training and basic content of our proposed experimental intervention will be standardized, the content of individual sessions will require some flexibility, consistent with the practice of motivational interviewing. To maintain an appropriate balance between individual “tailoring” and use of a "standardized" intervention, several features have been incorporated. Participants will have a standardized number of sessions according to a set schedule. Counselors will use the standardized manual to conduct the experimental intervention. To measure achieved fidelity of the experimental intervention implemented, counselors will complete a quantitative assessment at the end of each experimental counseling session that will capture the topics reviewed and goals set. To compliment the quality assurance measures completed by the counselors, participants in the experimental group at the end of each session will be asked to indicate topics discussed as part of their post-session quantitative assessment. A person separate from the counselor will administer this questionnaire. These data together with the summaries provided by the counselors

at the end of each session will be analyzed to determine the extent to which the experimental intervention sessions were implemented as planned.

Counselors and participants in the standard-of-care arm will also document topics covered during the standard-of-care counseling sessions (enrollment/baseline and weeks 1 and 2).

In addition, provider/participant observation of control and intervention sessions will be conducted periodically by a supervisor. The purpose of the observations is to serve as external quality control to determine fidelity to the intervention and provider/participant rapport.

5.0 STUDY PROCEDURES

An overview of the visit procedures schedule is provided in Appendix I. Detailed instruments to guide standard study procedures across sites will be provided in the Study Specific Procedures (SSP) Manual, which will be provided to each site prior to study implementation. Presented below is additional detail on visit-specific study procedures.

Note: Study Procedures, including delivering the intervention and completing the assessments, may be conducted off-site at a location such as the participant's home or workplace if requested by the participant and agreed to by study staff. In so doing, study staff will take measures to ensure discretion and confidentiality. It is anticipated that this option will be used infrequently.

Note: Data obtained as part of CHAVI 001 assessments will be used in HPTN 062.

5.1 Screening/Enrollment (Day 0)

Screening and enrollment will most likely take place on the same day but may take place on different days based on the preferences of the study participants and availability of study staff. Individuals will be informed of their HIV test results and receive standard HIV post-test counseling for risk reduction as part of CHAVI 001. Thereafter participants will be introduced to HPTN 062. Only individuals with AHI who are enrolled in CHAVI 001 are eligible for screening for HPTN 062. Written informed consent will be obtained for HPTN 062 before study procedures are initiated. Participants who provide consent will be enrolled in the study and administered a baseline behavioral assessment. Participants will then be randomized to the experimental arm or control arm. Individuals randomized to the experimental arm will participate in the introductory intervention session (session 1), ideally on this day; however, due to time constraints the introductory intervention session may be scheduled later.

At the screening/enrollment visit, participants will receive the following:

Screening/Enrollment and Baseline Activities

- Standard HIV post-test counseling
- 062 Study Informed Consent
- Randomization
- Participant Pre- and Post-session Assessments
- Experimental Intervention Session 1: Introductory Session (experimental arm only)

Note: To ensure that individuals are in the acute/early phase when the intervention begins, those who report to the clinic 45 days after laboratory detection of AHI will not be eligible for

screening or enrollment. This is consistent with CHAVI 001 enrollment eligibility criteria for AHI.

Counselors will complete the Counselor Post-Session Assessment after the enhanced counseling session at baseline for the experimental arm, and after the standard-of-care counseling session at baseline for control arm.

5.2 Follow-up Visits at Weeks 1, 2, 4 and 8

For the purposes of scheduling intervention sessions and follow-up visits, Day 0 will be considered the day of enrollment and visits will be scheduled from this date. When possible, HPTN 062 visits will be scheduled at the same time as CHAVI 001 visits. All HPTN 062 visit time points are consistent with those for CHAVI 001, with the following exceptions there may be an additional visit scheduled between enrollment and week 2 for the second intervention session and assessments for participants in the experimental arm and there will be an additional visit at week 20 for a behavioral assessment for participants in each arm.

Note: The ideal intervention schedule is: the first session delivered on the day of enrollment, the second session delivered 3 days after the first session, the third session delivered at week 1 (day 7 after enrollment), and the fourth session delivered at week 2 (14 days after enrollment). However, flexibility is allowed to accommodate participants' schedules, with the goal of completing all four sessions in the first two weeks after enrollment. The fifth session, which is the booster session, will be at week 8.

Note: Participants in the experimental arm who do not present to the clinic or will be unable to present for an intervention session within the target visit window will be offered an opportunity to make up that session. Participants can complete more than one session during a visit.

Participants will receive the following:

- Participants Pre and Post session Assessments
- Standard HIV risk-reduction counseling as part of CHAVI 001 (weeks 1, 2, 4 and 8)
- Experimental Intervention Sessions 2-4 (experimental arm only, between enrollment and week 2/day 14)
- Booster Session (experimental arm only, week 8)
- Qualitative Interview (both arms, weeks 2 and 8)

  Note: As part of CHAVI 001 participants in both arms will receive standard HIV risk-reduction counseling at week 3.

Counselors will complete the Counselor Post-Session Assessment after the enhanced counseling session at Session 2 (day 3), Session 3 (week 1), Session 4 (week 2), Session 5 (week 8) for the experimental arm, and after the standard-of-care counseling session at week 1 and week 2 for the control arm.

5.3 Follow-up Visits at Weeks 12, 16, 20 and 24

Follow-up visits at weeks 12, 16 and 20 and 24 may take place within the period extending from 2 weeks prior to the target date to 2 weeks after the target date. The visit schedule and visit windows are consistent with CHAVI 001.

Participants will undergo the following:

- Standard HIV risk-reduction counseling as part of CHAVI 001 (weeks 12, 16, and 24)
- Behavioral Assessment (weeks 12, 16, 20 and 24)
- Qualitative Interview (weeks 12 and 24)

5.4 Language of Interview and Translation Process

The research team will develop questionnaires and the consent form in English and have them translated and back-translated into the local languages. Interviews will be conducted in the language chosen by the participant.

5.5 Methods to Improve Self-Reported Sexual Behavior

Interviews will be conducted in a private office at the study sites or at another location chosen by the participant. Interviewers will be trained counselors or public health professionals with experience in conducting interviews on sensitive behaviors. In addition, interviewers will undergo training as part of the study on good interviewing techniques and on the questionnaire itself. The training will emphasize the importance of asking questions in a neutral tone; collecting data without bias, judgment, or subjectivity; building rapport; being an active listener; making the participant feel comfortable; and ensuring privacy and confidentiality throughout the study.

6.0 ADVERSE EVENTS AND SOCIAL HARMS

Because this study includes no biomedical intervention or study product, standard adverse-event reporting will not be undertaken and no adverse-event data will be collected on case report forms (CRFs) for entry into the study database. However, in accordance with 45 CFR 46, unanticipated problems or serious adverse events that are judged to be related or possibly related to study participation will be documented and reported to the IRB/ECs according to their individual requirements and to the DAIDS Medical Officer and the Office for Human Research Protections (OHRP). This reporting will be performed according to the timelines and definitions included in the SSP manual and the guidelines provided at www.hhs.gov/ohrp/policy/AdvEvntGuid.htm.

Given the potential for harm that surrounds partner HIV notification/disclosure and referral, there are potential social harms that could occur as a result of participation in this study, if a participant chooses to inform their sexual partners. For example, while only one situation of actual harm was reported, CHAVI 011 participants expressed the concern that a female with AHI might not disclose her status to her male partner and refer him for counseling and testing because she fears her partner will respond aggressively. Participants also said that females could be accused of infecting their male partners if the females learn and disclose their status first, and that male partners might end the relationship. For these reasons, in order to limit social harms, counselors will explore the possibility of social harms with each participant who wishes to disclose or who wishes for a counselor to notify her/his partner(s), prior to the event.
If a participant feels that a social harm is a possibility, counselors will discuss what the participant could do to prevent the harm from occurring (e.g., change the behavior; ask counselors to speak with partners). All subjects will also be reminded of the importance of reporting problems to study staff between regularly scheduled visits, and instructed on how to contact study staff should problems occur during intervals between visits.

Social harms judged to be significant by the Investigator of Record will be reported to the appropriate CHAVI 001 staff and the DAIDS Medical Officer according to the procedures stated in the SSP manual

and to relevant IRB/ECs at least annually, or according to each IRB/EC's requirements. Social harms that are determined to be unanticipated problems involving risks to the subjects will be reported to the IRBs/ECs as they are identified.

7.0 STATISTICAL CONSIDERATIONS

7.1 General Design

This is a multi-site, two-arm, randomized, prospective study designed to evaluate the feasibility and acceptability of a counselor-delivered behavioral intervention for individuals diagnosed with acute and early HIV infection. The intervention aims to reduce risk behaviors among individuals in the acute and early phase HIV infection. A target of 46 individuals who have been diagnosed with acute HIV infection and are participating in CHAVI 001 will be enrolled at multiple sites and randomized in a 1:1 ratio to receive an experimental four-session series of individual counseling and a fifth, booster session. All participants will receive standard risk-reduction counseling provided through CHAVI 001. Participants will be followed for 6 months from enrollment. Sexual behavior data will be collected longitudinally. Feasibility and acceptability of the intervention will be assessed through session attendance records and interviews with participants. Interviews will also be conducted with the counselors who complete the sessions to assess perceived effectiveness of the intervention as well as topics related to the content, delivery, and format of the intervention.

7.2 Study Endpoints

Data from the quantitative assessments and qualitative interviews will contribute to the primary and secondary endpoints.

7.2.1 Primary Endpoints

Feasibility:

- Proportion of participants in the experimental arm who receive the first four intervention sessions in the first 2 weeks after being informed of their diagnosis
- Proportion of participants in both arms who receive their respective counseling sessions between enrollment and week 8
- Proportion of participants in the experimental arm who receive the first four intervention sessions at any time point after enrollment

Acceptability:

- Measures related to the intervention format, content, and perceived effectiveness, as follows:

Format

Primary

- Acceptability of when to start the intervention (at the time the participant is informed of the diagnosis or later)
- Acceptability of session length
- Acceptability of number of sessions
- Acceptability of session spacing

Secondary

- Acceptability of individual versus group sessions

- Acceptability of characteristics of counselor

Content

- Perceived satisfaction of each session

Perceived effectiveness of session

- Perceived helpfulness of each session

7.2.2 Secondary Endpoints

Intervention effectiveness:
Number of self-reported unprotected sex acts

Sexual behavior and perception:

- Number of new sexual partners
- Number of concurrent sexual partners
- Participant perceptions of infectiousness

Information will be collected on potential mediators of sexual behavior, even though those numbers will be small. Examples of the outcome, mediators, and moderators that may be measured are described in Table 4.

Table 4 – Outcomes and examples of potential scales to measure mediators and moderators

Domain	Name of Variable	Variable Description	Potential Time Points Administered	Measure
**Outcomes – Effectiveness**	Number of unprotected sex acts in x period	# episodes of unprotected intercourse (vaginal/anal) in the last week/month only	Baseline and 2, 4, 8, 12, 16, 20, 24 weeks	
**Potential Mediators/ Moderators**	Motivation to avoid unsafe sex	9-item scale: HIV risk-avoidance intention	Baseline and 2, 4, 8, 12, 16, 20, 24 weeks	Kalichman, 96
	Self-efficacy	6 items, 4 point response set	Baseline and 2, 4, 8, 12, 16, 20, 24 weeks	Kalichman, 06
	Perceived infectiousness	Perceptions of infectiousness related to stage of infection	Baseline and 2, 4, 8, 12, 16, 20, 24 weeks	Adapted from Kalichman, 2002
	Ambivalence	16-item, 4-point scale on ambivalence related to safer sex behavior	Baseline and 4, 8, 12, 16, 24 weeks	Thompson, 1995
	Substance use	Drug/alcohol use and frequency	Baseline and 4, 8, 12, 16, 24 weeks	
	Social support	15-item, 4-point scale of available support	Baseline and 8, 24 weeks	Kalichman, 99
	Depression	CES-D, 20-item scale	Baseline and 8, 24 weeks	CES-D
	Coping	Ways of coping scale	Baseline and 8, 24 weeks	Fleischman, 00
	Sexual coercion/violence	Experience of forced sex	Baseline and 8, 24 weeks	Dunkle, 04

7.3 Sample Size and Accrual

A total of approximately 46 individuals diagnosed with acute and early HIV infection will be enrolled at two or more sites over a period of approximately 24 months. Individuals will be enrolled in HPTN 062 as soon as possible after being diagnosed with AHI through CHAVI 001. Each arm will have a total of approximately 23 participants. The sample size is limited by the expected low incidence rate for AHI (based on data available from the ongoing studies). For example, in the CHAVI 001 study, about 1 percent of subjects screened have been identified as acutely infected with HIV. Therefore, in the same or similar study settings, approximately 4600 individuals will need to be screened through other studies or programs to identify 46 individuals with AHI who may be eligible for and willing to participate in HPTN 062. Due to the limited sample size expected for this study (n=46), all sample-size calculations assume data will be pooled across sites.

Note: A minimum of 46 participants is targeted; however, the final number may be higher or lower, depending on the rate of accrual in CHAVI 001 during the HPTN 062 enrollment period of approximately 24 months.

Since the sample size is not large (about 23 per arm), the exact binomial confidence interval is calculated. Table 5 shows the expected precision (i.e. 95 percent CI) for estimates of proportions that will be obtained from this study as a function of the total sample size and the observed proportion. For example, for the proportion of participants who complete 4 intervention sessions in the first 2 weeks after enrollment, if the sample size is 23 in the experimental arm and the observed proportion is 0.8, the 95 percent CI for the proportion will be (0.58, 0.94),. Proportions of adherence will be estimated separately for each arm.

Table 5 - 95% CI for sample proportion as a function of observed proportion and sample size, e.g. 95% CI =(0.58, 0.94)**, if p = .8 and n = 23**

Sample Size per arm, Pooling All Sites					
**Observed Proportion**	20	23	30	40	46
0.5	(0.27, 0.73)	(0.29, 0.71)	(0.31, 0.69)	(0.34, 0.66)	(0.35, 0.65)
0.6	(0.36, 0.81)	(0.38, 0.80)	(0.41, 0.77)	(0.43, 0.75)	(0.45, 0.74)
0.7	(0.46, 0.88)	(0.48, 0.87)	(0.51, 0.85)	(0.53, 0.83)	(0.55, 0.83)
0.8	(0.56, 0.94)	(0.58, 0.94)	(0.61, 0.92)	(0.64, 0.91)	(0.66, 0.90)
0.9	(0.68, 0.99)	(0.70, 0.98)	(0.73, 0.98)	(0.76, 0.97)	(0.78, 0.97)

Note that this study is not powered to evaluate the efficacy of the intervention effect. The sample size is limited by the availability of subjects diagnosed with the AHI. Table 6 displays the power of detecting efficacy for a range of hypothesized true values of the effect size at the 0.05 significance level. The powers in the table were estimated by a simulation of 10,000 trials under the assumption that the numbers of self-reported unprotected sex acts in the control and intervention arms are distributed as a negative binomial random variable with mean mu and variance phi*mu (phi is the over-dispersion parameter). The negative binomial distribution is a mixture of Poisson distributions with mean distributed as a Gamma distribution with scale parameter, (1 - p)/p, and shape parameter, r. So the mean mu is equal to r*(1 - p)/p and variance r*(1 - p)/p^2 with phi=1/p. For phi=1, the mean and the variance are the same, such that the number of self-reported sex acts are simply Poisson distributed with no over-dispersion. Figure 2 gives the histogram of simulated sex acts under different negative binomial distributions. Simulations were done for three levels of over-dispersion: none (phi=1), mild (phi=2), and severe (phi=5). The powers were estimated based on a cross-sectional study, i.e., a single observation per subject. Higher power will likely be obtained for this study because of longitudinal data being collected. Although the study is not sufficiently powered, if the effectiveness of the intervention is truly high, a sample size of 23 in each group would yield 92 percent (62 percent) power to detect the mean difference of 2 in self-reported unprotected sex acts in two groups assuming over-dispersion parameter of 2 (5) if the mean of sex acts is 1 in the intervention group and 3 in the control group. .

Figure 2. Histogram of sex acts from simulated data under negative binomial distributions each with different mean (mu) and over-dispersion parameter (phi).

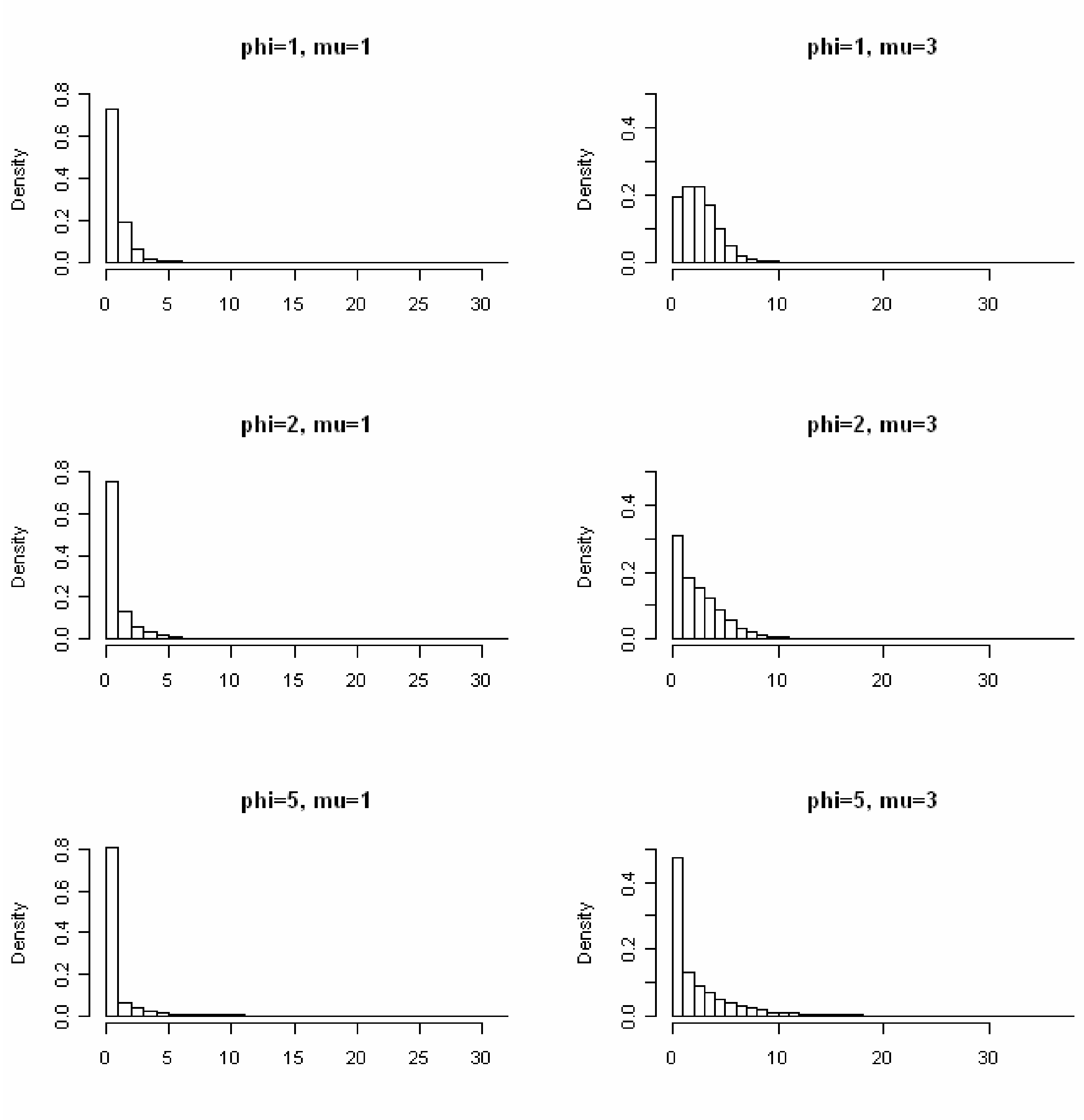

Table 6 - Power to detect a significant difference in self-reported unprotected sex acts between control and intervention groups. The type I error was set at 0.05 for a two-sided test.

Sample Sizes per Arm Pooling All Sites											
			phi=1			phi=2			phi=5		
**Control group mean (x1)**	**Intervention group mean (x2)**	**Mean difference (x1-x2)**	**20**	**23**	**30**	**20**	**23**	**30**	**20**	**23**	**30**
1.5	0.5	1.00	0.90	0.93	0.98	0.63	0.68	0.79	0.39	0.42	0.50
2.0	0.5	1.50	0.99	1.00	1.00	0.87	0.90	0.96	0.56	0.61	0.70
2.5	0.5	2.00	1.00	1.00	1.00	0.96	0.97	1.00	0.70	0.75	0.84
2	1	1.00	0.74	0.80	0.89	0.45	0.50	0.61	0.27	0.29	0.34
2.5	1	1.50	0.95	0.98	0.99	0.72	0.77	0.87	0.44	0.46	0.55
3	1	2.00	1.00	1.00	1.00	0.88	0.92	0.97	0.57	0.62	0.71
2.5	1.5	1.00	0.60	0.67	0.79	0.35	0.39	0.49	0.21	0.22	0.27
3.0	1.5	1.50	0.89	0.93	0.98	0.60	0.65	0.77	0.34	0.37	0.44
3.5	1.5	2.00	0.98	0.99	1.00	0.80	0.85	0.93	0.48	0.52	0.61

7.4 Randomization

Participants who meet the study's eligibility criteria will be randomly assigned in a 1:1 ratio either to the control or the experimental arm. The randomization will be stratified by study site. The randomization will be conducted by the HPTN SDMC.

7.5 Blinding

This is an unblinded study. Participants are not blinded due to the nature of the intervention.

7.6 Analysis Plan

7.6.1 Primary Analysis

7.6.1.1 Analysis of Feasibility

Feasibility will be evaluated by the following:

1.) The proportion of enrolled participants in the experimental arm who attend the first 4 intervention sessions in the first 2 weeks after enrollment out of all individuals randomized to the experimental arm. An estimate of the proportion is given by

$$P = \frac{A}{N}$$

where N is the total number of participants enrolled in the experimental arm and A is the number of individuals who attend the first 4 intervention sessions in the first 2 weeks among those N participants. Since the sample size is not large (about 23 per arm), the exact binomial confidence interval will be calculated for the estimated proportion.

2.) The proportion of enrolled participants in the experimental arm who attend the 4 intervention sessions and the fifth, booster session out of all individuals randomized to the experimental arm, and compared to the proportion of individuals who attend their CHAVI 001 visits at weeks 1, 2, and 8. The two-sided Fisher exact test will be used to test 2 proportions.

3.) The proportion of enrolled participants in the experimental arm who attend the first 4 intervention sessions at any time point after enrollment out of all individuals randomized to the experimental arm. This analysis will include participants who attended all of the first four intervention sessions but not necessarily within the 2 week period after enrollment. An estimate of the proportion is given by

$$P = \frac{A}{N}$$

where N is the total number of participants enrolled in the experimental arm and A is the number of individuals who attend the first 4 intervention sessions at any time point among those N participants.

Feasibility endpoints will be measured using session attendance records. Data from the quantitative assessments and qualitative interviews will not contribute to the analysis of the feasibility endpoints.

7.6.1.2 Analysis of Acceptability

Data from the quantitative assessment and qualitative interviews will contribute to the analysis of the acceptability endpoints. Standard descriptive statistics, such as means, medians, variance, and interquartile ranges for continuous variables and proportions for binary variables will be used to summarize the format, content, and perceived effectiveness. For categorical variables, the numbers and the proportions will be tabulated. Analysis of the qualitative data related to acceptability is described below is Section 7.6.3 Qualitative Analysis.

7.6.2 Secondary Analysis

Standard descriptive statistics, such as means, medians, variance, and interquartile ranges for continuous variables and proportions for binary variables will be used to summarize the characteristics of sexual behavior, depression, substance use, and motivation for safe sex at baseline and/or subsequent study visits in both study arms. For categorical variables, the numbers and the proportions will be tabulated. **Particularly, for each participant, the pre-post difference at each visit and the difference between current and previous visits will be visually explored to look for any trend using a single-case analysis approach. A case study and mixed-methods (e.g. using both qualitative and quantitative data) approach will also be considered in the single-case situation to better understand the intervention effect among particular study subjects. Some nonparametric smoothing will be considered to fit the longitudinal curves to further explore the trends.**

To assess the efficacy of the intervention on sexual behaviors, such as self-reported unprotected sex acts with the intervention, Poisson regression will be used for count endpoints based on generalized estimation equations (Liang, Zeger, et al., 1986). Effects of the intervention on sexual behaviors at each time point will be assessed to see whether there are differences in behaviors after the intervention or at any time. In addition, to determine whether there are changes in behavior after viral loads have stabilized, behaviors will be compared before and after 12 weeks post-diagnosis. A robust variance estimator will be used to correct for correlation among the longitudinal measures at baseline and post-intervention follow-up visits. If over-dispersion exists, quasi-Poisson or negative binomial model will be used. A Wald test will be used to test whether the intervention has significant effect on reducing sexual risk behaviors compared to the control arm. It will be a two-sided test at a significance level of 0.05. **Given all the participants are acute or early HIV-infected, it is possible that there may be few, if any, unprotected sex acts reported. Therefore, the negative binomial assumption might not hold and a non-parametric approach will be considered. Specifically, unprotected sex acts will be categorized into 2 or 3 groups. At each visit, a Fisher exact test will be used to test proportions difference for two arms and exact p-values will be calculated.**

The impact of mediator variables (Table 4) on mediating the effects of the intervention on self-reported unprotected sex behaviors will be assessed. Multivariate analysis of variance (MANOVA) will be used to determine the relationship between intervention and changes in the mediator variables with repeated measures over the visits if the normality assumption holds. For the categorical variables, Chi-squared tests will be used to test independence between intervention and mediator variables at each visit.

7.6.3 Qualitative Data Analysis

Qualitative data will be collected on the acceptability of the intervention, intervention effectiveness, and sexual behavior and perception in order to help answer the primary and secondary objectives. The same analysis techniques will be applied to all qualitative data. Data analysis will begin with site staff transcribing and translating each audio taped interview. Sites may choose to simultaneously transcribe and translate the interview text from the original language to English or they may choose to transcribe the interview text in to the local language used during the interview and then translate it into English. A standardized transcription process will be followed.

As soon as possible after the interviews are conducted, transcript text will be read and re-read carefully by designated site staff and/or Protocol Co-chairs or their designees to: (1) become familiar with each individual situation; (2) identify text that may be unclear due to differences in the cultural context; (3) point out areas in which interviewing and transcription techniques could be improved; and (4) identify recurrent themes and areas for future probing.

Analysis will begin by creating codes for retrieving text from interview transcripts related to acceptability, intervention effectiveness, and sexual behavior and perception. Data-derived codes related to the overall objectives may also be used if identified through inductive coding and retrieving. The Protocol Chairs, site PIs, and study analysts will determine a coding frame to be used based on the qualitative questionnaires and the first few interviews available for analysis. NVIVO or another qualitative software program will be used to organize all qualitative data and prepare it for analysis. Procedures will be put into place to check for inter-coder reliability. Discrepancies will be noted, discussed, and resolved by the Protocol Co-chairs, site PIs, and study analysts. The resolution of such discrepancies will be amended to the existing coding

framework. Once all the transcripts have been coded, textual coding reports will be produced. Data display matrices will be developed to examine each code in detail for sub-themes and patterns across the interviews, followed by data summaries.

As in most studies where qualitative interviewing is used, questions may be modified, added to, or deleted from the qualitative questionnaires during data collection based on information that is learned from conducting and reading the transcripts from the previous interviews. We will also modify the questionnaire as appropriate after the training practice sessions. Grounded in qualitative methodology, this approach allows researchers to explore in more detail themes that emerge during data collection, and improves understanding of the overall issues. These modifications to the question guides will be made after approval from the ethics committees (EC)/institutional review boards (IRB) and will not be resubmitted; however, *only* probes and questions that are related to the overall topics described in this protocol and similar to the original questions will be added. Any changes made to the question guides that are beyond the topics described here will be resubmitted for EC/IRB review and approval prior to use. Limited changes will be made to questions that assess the acceptability of the intervention so comparable questions are asked to all participants.

8.0 HUMAN SUBJECTS CONSIDERATIONS

8.1 Ethical Review

This protocol and the informed-consent form template contained in Appendix II -- and any subsequent modifications -- will be reviewed and approved by the HPTN Scientific Review Committee and DAIDS Prevention Science Review Committee with respect to scientific content and compliance with applicable research and human subjects regulations.

The protocol, site-specific informed consent forms, participant education and recruitment materials, and other requested documents -- and any subsequent modifications -- also will be reviewed and approved by the ethical review bodies responsible for oversight of research conducted at the study site.

Subsequent to initial review and approval, the responsible IRBs/ECs will review the protocol at least annually. The Investigator will make progress reports to the IRBs/ECs at least annually, and within three months of study termination or completion. These reports will include the total number of participants enrolled in the study, the number of participants who completed the study, all changes in the research activity, and all unanticipated problems involving risks to human subjects or others. Study sites will submit documentation of continuing review to the DAIDS Protocol Registration Office in accordance with the current DAIDS Protocol Registration Policy and Procedure Manual which can be found on the following website: http://rcc.tech-res.com/

8.2 Informed Consent

Written informed consent will be obtained from each study participant and also from counselors who deliver the intervention, using different consent forms from CHAVI 001. Each study site is responsible for developing study informed consent forms for local use (based on the templates in Appendix IIA and IIB) that describe the purpose of the study, the procedures to be followed, and the risks and benefits of participation, in accordance with all applicable regulations. The HPTN Coordinating and Operations Center (CORE) will work closely with the study site to ensure that the informed consent forms are translated into local languages, and that the accuracy of the translation is verified by performing an independent back-translation.

Literate participants will document their provision of informed consent by signing their informed consent form. Non-literate participants will be asked to document their informed consent by marking their informed consent form (e.g., with an X, thumbprint, or other mark) in the presence of a literate third-party, impartial witness. (Further details regarding DAIDS requirements for documenting the informed consent process with both literate and non-literate participants are provided in the *Requirements for Source Documentation in DAIDS Funded and/or Sponsored Clinical Trials*.) Any other local IRB/EC requirements for obtaining informed consent from non-literate persons also will be followed.

Participants will be provided with copies of their informed consent forms if they are willing to receive them.

It will be stressed with each potential participant with AHI that participation in this study is completely voluntary and is independent from her/his participation in CHAVI 001. Potential participants will be told that if they choose not to participate, there will be no penalty; they can continue to participate in other research studies, including CHAVI 001, or programs at the site and there will be no changes in the services they may receive at the clinic where they were contacted about this study.

Counselors who deliver the intervention will be asked to sign a separate consent form indicating that they agree to be interviewed approximately three times to capture their perceptions of the acceptability and perceived impact of the intervention.

8.3 Risks

This is an intervention study involving extensive individual counseling. No biological specimens will be collected and there is no biomedical intervention. Participants may become embarrassed, worried, or anxious when completing their HIV risk assessment and/or discussing sexual behaviors and other issues related to the prevention of HIV, such as partner disclosure, during the intervention. The counselors who deliver the intervention are trained to help participants deal with these feelings. It will also be emphasized that participants may feel free not to answer a question asked during any assessment and to terminate any interview or leave the behavioral intervention sessions at any time.

Although study sites will make every effort to protect participant privacy and confidentiality, it is possible that participants' involvement in the study could become known to others and that social harms may result (i.e., because participants could become known as HIV-infected or at "high risk" for HIV infection or “high risk” for HIV transmission). For example, participants could be treated unfairly or discriminated against, or they could have problems being accepted by their families and/or communities. Finally, state or local officials may require study sites to report the names of study participants who disclose (in the context of a counseling session or in response to a questionnaire) an intention to harm self or others, current or recent child abuse, or current or recent statutory rape.

The risks will be described in the study consent form and will be thoroughly discussed with all potential study participants prior to participation.

CHAVI 011 data suggest that risks of violence from partner notification may be extremely limited; however, participants described the termination of relationships as a result of disclosure and many expressed fears of their partner’s reactions and fears of discrimination from family/friends/community as a result of disclosure. These findings heavily informed the partner notification component of the HPTN 062 curricula. To reduce possible harm as a result of

partner notification, partner notification is an optional component of the experimental intervention. For participants who chose to disclose, they select the method which is most comfortable to them. If a participant chooses to notify partners, a full discussion of the potential risks will be discussed during the intervention session.

If requested by the participant, study staff may contact the partners of any participant reporting harm as a result of disclosure. For participants who wish for staff to notify their partner(s), only individuals who provide verbal consent for providers to contact partners will have partners contacted. Potential harm will be further minimized during a provider notification process by not disclosing the name or any personal information about the sex partner to those who are contacted by the study staff, unless the participant consents to providing this information. Staff will document in the participant's clinic notes that the participant gave permission to contact her/his sexual partner(s) and whether or not staff can disclose the participant's name to the partner(s). Additionally, when contacting the partner(s), study staff will first confirm the identity of the partner using the partner's name and other key identifying features provided by the study subject before providing any notification information. Study staff will also never leave study documents with a friend/family member to pass on to the partner.

Currently, there are no local or national requirements for disclosure of HIV status in Malawi. However, if local or national requirements are established during the course of the study then they will be followed.

There are no anticipated risks for counselors who participate in the assessments, as they are participating in this study in a professional role.

8.4 Benefits

While there may be no or limited benefits to participants with AHI who take part in this research, participating in the prevention intervention may have some psychosocial benefits. For example, participants who take part in the prevention intervention may cope with their status better, because participation provides an opportunity to discuss one's feelings. Participants and others may benefit in the future from information learned from this study. Specifically, information learned may lead to the development of an effective prevention program that may help reduce the spread of HIV infection.

8.5 Incentives

Pending IRB/EC approval, participants with AHI who participate in the behavioral intervention will be compensated for their time and effort in this study and/or be reimbursed for travel to study visits and time away from work. Site-specific reimbursement amounts will be specified in the study informed-consent forms.

8.6 Confidentiality

All study-related information will be stored securely at the study site. All participant information will be stored in locked file cabinets in areas with access limited to study staff. All reports, audiotapes, study data-collection forms, and process and administrative forms will be identified by a coded number only to maintain participant confidentiality. The use of participant identifiers on study records will comply with the DAIDS policies for Source Documentation and Essential Documents. All local databases will be secured with password-protected access systems. Forms, lists, logbooks, appointment books, and any other listings that link participant identification numbers to other identifying information will be stored in secured files in an area with limited access.

The confidentiality of study participants with respect to their sexual partners will be protected to the extent possible. Partner notification is not a requirement of this study; however, it will be encouraged as part of the intervention. Existing operating procedures will be followed to protect the confidentiality of individuals with AHI by never revealing the potential source of partner information, unless permission is received by the participant, as described above. Only in situations where study staff believes a sexual partner of a study participant with AHI is in immediate physical danger will they potentially breach confidentiality to protect the sexual partner.

Contact information from participants with AHI who take part in the prevention-intervention pilot will be collected in order to follow up with those who miss sessions. Participants will be contacted only by means (e.g. cell phone, home visit) to which they agree.

Participants' study information will not be released without the written permission of the participant, except as necessary for monitoring by the NIAID and/or its contractors; representatives of the HPTN CORE, SDMC, and/or Network Lab (NL); government and regulatory authorities, and/or site IRBs/ECs.

8.7 Study Discontinuation

The study may be discontinued at any time by the sponsors NIAID, OHRP, the HPTN, CHAVI, or US or in-country government or regulatory authorities.

9.0 LABORATORY SPECIMENS AND BIOHAZARD CONTAINMENT

There will be no laboratory assessments as part of this protocol.

10.0 ADMINISTRATIVE PROCEDURES

10.1 Study Activation

Following ethical review and approval, each participating site will complete the DAIDS protocol registration process as described in the DAIDS Protocol Registration Manual. The HPTN CORE, SDMC, and NL staff will work closely with each site to ensure completion of this and all other study-specific site-activation requirements, as detailed in the HPTN Manual of Operations and the SSP Manual. Upon successful protocol registration and completion of all other study-specific site-activation requirements, the CORE will issue a study-activation notice to the site. Implementation of the study may not proceed prior to receipt of this written notification.

10.2 Study Coordination

Study implementation will be directed by this protocol as well as the SSP manual. The SSP manual will outline procedures for conducting study visits, data and forms processing, and other study operations.

Study case report forms (CRFs) and qualitative questionnaires will be developed by the study team and HPTN SDMC. CRF data will be transferred to the HPTN SDMC, entered, and cleaned using the SDMC data management system. Quality control reports and queries will be routinely sent back to the site for verification and resolution. Qualitative data collected through in-depth interviews will be managed and analyzed by members of the protocol team at Family Health International in collaboration with the study team and sites.

Close cooperation between the study Investigator, NIAID Medical Officer, Protocol Specialist, Biostatistician, SDMC Project Managers, and other study team members will be necessary in order to track study progress, respond to queries about proper study implementation, and address other issues in a timely manner. Rates of accrual, adherence, and follow-up will be monitored closely by the study team. These rates also will be evaluated by representatives of the HPTN CORE and SDMC on a regular basis.

10.3 Study Monitoring

On-site study monitoring will be performed in accordance with DAIDS policies. Study monitors will visit the site to:

- Verify compliance with human participants and other research regulations and guidelines
- Assess adherence to the study protocol, study-specific procedures manual, and local study operating procedures
- Confirm the quality and accuracy of information collected at the study site and entered into the study database.

Site investigators will allow study monitors to inspect study facilities and documentation (e.g., informed consent forms, clinic and laboratory records, other source documents, case report forms, transcripts), as well as observe the performance of study procedures. Investigators also will allow inspection of all study-related documentation by authorized representatives of the CHAVI, HPTN CORE, SDMC, NL, NIAID, and US and in-country government and regulatory authorities. Acceptable source documentation for each site will be specified before the study begins. A site-visit log will be maintained at the study site to document all visits.

10.4 Protocol Compliance

The study will be conducted in full compliance with the protocol. The protocol will not be amended without prior written approval by the Protocol Chair and NIAID Medical Officer. All protocol amendments must be submitted to and approved by the relevant IRB(s)/EC(s) prior to implementing the amendment except when necessary to protect the safety, rights, or welfare of participants or to eliminate apparent immediate hazards to participants.

10.5 Investigator's Records

The study site investigator will maintain and store in a secure manner complete, accurate, and current study records throughout the study. The investigator will retain all study records for at least three years after the completion of the study, unless directed otherwise by DAIDS. Study records include administrative documentation, including site-registration documents and all reports and correspondence relating to the study, as well as documentation related to each participant screened and/or enrolled in the study, including informed consent forms, locator forms, case report forms, notations of all contacts with the participant, and all other source documents. Qualitative data collected from in-depth interviews, including audiotapes, original transcripts, and English translations, will also be maintained along with other study records.

10.6 Use of Information and Publications

Publication of the results of this study will be governed by HPTN and CHAVI policies. Any presentation, abstract, or manuscript will be submitted by the Investigator to the HPTN Manuscript Review Committee, CHAVI Scientific Leadership Committee, and DAIDS for review prior to submission.

11.0 REFERENCES

Abu-Raddad LJ, Longini IM Jr. No HIV stage is dominant in driving the HIV epidemic in sub-Saharan Africa. *AIDS.* 2008;22(9):1055-61.

Colfax GN, Buchbinder SP, Cornelisse PG, Vittinghoff E, Mayer K, Celum C. Sexual risk behaviors and implications for secondary HIV transmission during and after HIV seroconversion. *AIDS* 2002;16(11)**:**1529-35.

Crepaz N, Lyles CM, Wolitski RJ, et al. Do prevention interventions reduce HIV risk behaviours among people living with HIV? A meta-analytic review of controlled trials. *AIDS* 2006;20(2)**:**143-57.

Fisher JD, Fisher WA, Cornman DH, Amico RK, Bryan A, Friedland GH. Clinician-delivered intervention during routine clinical care reduces unprotected sexual behavior among HIV-infected patients. *J Acquir Immune Defic Syndr* 2006;41(1)**:**44-52.

Fraser C, Hollingsworth TD, Chapman R, de Wolf F, Hanage WP. Variation in HIV-1 set-point viral load: epidemiological analysis and an evolutionary hypothesis. Proc Natl Acad Sci U S A. Oct 30 2007;104(44):17441-46.

Golin CE, Patel S, Tiller K, Quinlivan EB, Grodensky CA, Boland M. Start Talking About Risks: development of a Motivational Interviewing-based safer sex program for people living with HIV. *AIDS Behav* 2007;11(5 Suppl)**:**S72-83.

Halperin DT, Steiner MJ, Cassell MM, Green EC, Hearst N, Kirby D, Gayle HD, Cates W. The time has come for common ground on preventing sexual transmission of HIV.*Lancet.* 2004; 364(9449):1913-15.

Hollingsworth TD, Anderson RM, Fraser C. HIV-1 transmission, by stage of infection. *J Infect Dis*. 2008;198:687-93.

Jacquez JA, Koopman JS, Simon CP, Longini IM, Jr. Role of the primary infection in epidemics of HIV infection in gay cohorts. *J Acquir Immune Defic Syndr* 1994;7(11)**:**1169-84.

Koopman JS, Jacquez JA, Welch GW, et al. The role of early HIV infection in the spread of HIV through populations. *J Acquir Immune Defic Syndr Hum Retrovirol* 1997;14(3)**:**249-58.

Kretzschmar M, Dietz K. The effect of pair formation and variable infectivity on the spread of an infection without recovery. Math Biosci. Feb 1998;148(1):83-113.

Liang KY, Zeger SL. Longitudinal Data Analysis Using Generalized Linear Models. *Biometrika* 1986;73:13-22.

Pettifor A, Rees H, Kleinschmidt I, et al. Young people's sexual health in South Africa: HIV prevalence and sexual behaviours from a nationally representative household survey. *AIDS* 2005;19**:**1525-34.

Pilcher CD, Fiscus SA, Nguyen TQ, et al. Detection of acute infections during HIV testing in North Carolina. *N Engl J Med* 2005;352(18)**:**1873-83.

Pilcher CD, Joaki G, Hoffman IF, et al. Amplified transmission of HIV-1: comparison of HIV-1 concentrations in semen and blood during acute and chronic infection. *AIDS.* Aug 20 2007;21(13):1723-30.

Pilcher CD, McPherson JT, Leone PA, et al. Real-time, universal screening for acute HIV infection in a routine HIV counseling and testing population. *Jama.* 2002;288(2)**:**216-21.

Pilcher CD, Price MA, Hoffman IF, et al. Frequent detection of acute primary HIV infection in men in Malawi. *AIDS.* 2004;18(3)**:**517-24.

Pilcher CD, Shugars DC, Fiscus SA, et al. HIV in body fluids during primary HIV infection: implications for pathogenesis, treatment and public health. *AIDS* 2001;15(7)**:**837-45.

Pilcher CD, Tien HC, Eron JJ, Jr., et al. Brief but efficient: acute HIV infection and the sexual transmission of HIV. *J Infect Dis.* 2004;189(10)**:**1785-92.

Pinkerton SD. How many sexually-acquired HIV infections in the USA are due to acute-phase HIV transmission? *AIDS*. 2007;21(12):1625-29.

Pinkerton SD. Probability of HIV transmission during acute infection in Rakai, Uganda. *AIDS Behav.* 2008 (5):677-84

Shisana O, Simbayi L. Nelson Mandela/HSRC Study of HIV/AIDS South African National HIV Prevalence, Behavioural Risks and Mass Media Household Survey 2005. Cape Town: Human Sciences Research Council, 2005.

South African Department of Health. National HIV and Syphilis Prevalence Survey South Africa 2006. In: Health SADo, ed. Pretoria, 2007.

Stevens W, Akkers E, Scott L, et al. High Prevalence of Acute, undetected HIV infection in a Primary Care Clinic in South Africa. International AIDS Society Conference on Pathogenesis and Treatment July 2005, Rio De Janeiro, Brazil.

Wawer MJ, Gray RH, Sewankambo NK, et al. Rates of HIV-1 transmission per coital act, by stage of HIV-1 infection, in Rakai, Uganda. *J Infect Dis.* 2005;191(9)**:**1403-9.

Xiridou M, Geskus R, de Wit J, Coutinho R, Kretzschmar M. Primary HIV infection as source of HIV transmission within steady and casual partnerships among homosexual men. *AIDS* 2004;18(9):1311-20.

APPENDICES

Appendix I - Schedule of Visits and Procedures

	Screening/ Enrollment Day (0) (Session 1)	Day 3 (Session 2)	Week 1 (Session 3)	Week 2 (Session 4)	Week 3	Week 4	Week 8 (Session 5)	Week 12	Week 16	Week 20	Week 24
**Procedures**											
Study Informed Consent	**X**										
Post-test Standard Counseling Session	**X**										
Standard CHAVI 001 Counseling Session			**X**	**X**	**X**	**X**	**X**	**X**	**X**		**X**
Participant pre-session assessment	**X**		**X**	**X**		**X**	**X**	**X**	**X**	**X**	**X**
Participant post-Session assessment	**X**	**X (exp)**	**X**	**X**			**X (exp)**				
Counselor post-session assessment	**X**	**X (exp)**	**X**	**X**			**X (exp)**				
Qualitative Interview				**X**			**X**	**X**			**X**
Experimental Intervention Session (Experimental Arm Only)[1]	**Sessions 1, 2, 3, and 4**						**Booster Session**				
Counselor Observation[2]	X						X				

[1] The first four intervention sessions will be scheduled to occur from enrollment through the week 2 visit. Participants may undergo more than one session during a visit.

[2] Counselor Observation will be conducted randomly throughout the intervention period.

Appendix IIA - Sample Participant Enrollment Consent Form

HPTN 062
Feasibility and Acceptability Study of an Individual-Level Behavioral Intervention for Individuals with Acute and Early HIV-Infection
DAIDS ID: 10667

US National Institute of Allergy and Infectious Diseases (NIAID)
US National Institute on Drug Abuse (NIDA)
US National Institute of Mental Health (NIMH)
US National Institutes of Health (NIH)

Principal Investigator: *[insert name and contact information for the site PI]*

INTRODUCTION
You are being asked to take part in the research study named above. The person in charge of this study at this site is *[insert name of PI]*. This study is sponsored by the US National Institutes of Health (NIH).

You have been asked to participate in the research study because you have been recently diagnosed with acute HIV infection. Before you decide whether or not you want to take part in this research study, you need to know the purpose of the study, about the possible risks and benefits, and what will be expected of you if you decide to participate. This consent form provides information about the study. The study staff will also discuss the study with you. They will answer any questions that you have. After the study has been fully explained to you, you can decide whether or not you want to participate. This process is called informed consent. If you decide to participate in the study, you will be asked to sign this consent form or make your mark in front of someone. You will be offered a copy to keep.

VOLUNTARY PARTICIPATION
It is important that you know certain things:

- Your participation in the study is entirely voluntary.
- You may decide not to participate or to withdraw from the study at any time without losing the benefit of your standard medical care or other services.
- If you decide not to participate in this study, you can continue to participate in CHAVI 001 and other studies and can join another research study later, if one is available and you qualify.

PURPOSE OF THE STUDY
The purpose of the research study is to evaluate an enhanced HIV education and counseling program designed to help individuals with acute and early HIV infection reduce risk behaviors for HIV transmission. The study will determine whether the counseling program is feasible to implement and acceptable to participants. This study is taking place at several sites, including at least two in Africa. Approximately 40 to 60 people will participate in the study.

If you agree to participate in the study, the duration of your participation in this study will be for 6 months. Some information obtained from you and from your blood tests as part of the CHAVI 001 study in which you are participating will be used for this study also.

STUDY PROCEDURES

To find out if you are eligible for the study, the study staff will ask you some questions to determine if you are able and willing to participate. If you are eligible and you agree to take part in this study, you will be assigned to one of two groups. Which group you are in is decided by chance, like tossing a coin. About half of the participants will be in each group.

All individuals who enroll in this study will be scheduled for a visit today. Visits will be also scheduled at weeks 1, 2, 4, 8, 12, 16, 20 and 24. We will schedule these visits to coincide with your other CHAVI visits for your convenience. At these visits we will ask you questions about your sexual behavior and practices, your thoughts on the counseling you received, and other health-related topics. These questions may take about 45 minutes each visit. If you agree, part of your interview will be tape recorded so that we have an exact record of what you said. Your name will not be on the audiotape. The audiotape will be destroyed when data analysis is completed. You may still take part in the interviews even if you choose not to have your interview tape recorded.

Everyone in this study will receive counseling. Participants in one group will receive standard HIV risk reduction counseling as part of their CHAVI 001 visit. This is called the standard counseling group. This counseling is provided at every CHAVI 001 visit. If you do not join this study, you will still receive this risk reduction counseling as part of CHAVI 001.

Participants in the other group will receive standard HIV risk reduction counseling at each of their CHAVI 001 visits as well as additional HIV education and counseling sessions at some of their CHAVI 001 visits. This is called the enhanced counseling group. The enhanced counseling will include additional education about acute HIV infection, how to reduce the risk of transmitting HIV, and how HIV prevention strategies can be incorporated into your life. If you join this study described here and are placed in this group, you will be asked to attend these additional sessions mostly at the same time as your CHAVI 001 visits but you can come at other times if you like. These additional counseling sessions may last between 15 minutes to 1 hour, and will take place 5 times between enrollment and week 8 of your participation in CHAVI 001.

If you are in the enhanced counseling group, you will also be asked to attend another visit at about day 3 after enrollment for counseling. At some of your visits, you will be asked questions about your sexual behavior and practices, issues related to being diagnosed with HIV and your thoughts on the additional counseling you received and topics discussed during the sessions. In order to ensure quality of the sessions, some of the counseling sessions may be observed by a study team supervisor. If you do not want your session observed you have the right to refuse.

We would also like to document what was discussed at your post-test counseling session prior to joining this study, with your permission. The topics will be documented by the CHAVI 001 counselor.

Participants in both groups will be counseled at each visit about how to prevent passing HIV to other people and how to take care of their health. Participants will be encouraged to disclose their HIV status to their partners, although this is not a required component of the program. All of the sessions will be done in private.

RISKS AND DISCOMFORTS
We will make every effort to protect your privacy and confidentiality while you are in this study. However, it is possible that you could have problems if people learn that you took part in this study or if the information you provide becomes known to others. You may be treated unfairly by others, including your family and community.

Some of the sessions or interviews could make you feel uncomfortable or embarrassed. You may refuse to answer any question and you can leave any session or interview at any time. If any of the topics discussed in the session or interview upset you, we can refer you to a counselor with whom you can talk further.

If you choose to notify your partner, the counselors will discuss the risks with you during your counseling sessions.

POTENTIAL BENEFITS
You may receive no direct benefit from this research study. However, you may find it beneficial to discuss how HIV prevention strategies can be incorporated into your life and to talk with study staff about your health. Also, the information you provide us will be used to improve the program and may help others in the future.

REASONS WHY YOU MAY BE WITHDRAWN FROM THE STUDY WITHOUT YOUR CONSENT
The study staff may need to take you off the study early without your permission if:
- The research study is stopped or cancelled.
- You are not able to attend the study visits or follow the procedures required by the study.
- If the study staff believes that is it unsafe for you to continue in the study for any reason.
- Other administrative reasons

NEW FINDINGS
You will be told of any new information learned during the course of the study that might cause you to change your mind about staying in the study.

ALTERNATIVES TO PARTICIPATION
If you choose not to participate in this study or are not eligible to participate, you will still have access to the standard HIV-related counseling and services provided at this clinic and the staff will refer you to other health centers for additional services, if needed.

COSTS TO YOU
There will be no cost to you for study-related visits. For each scheduled visit that you attend during the study, you will be paid for your time and travel expenses. *[local amount to be specified in site specific consent.]*

CONFIDENTIALITY OF YOUR PERSONAL INFORMATION
All efforts will be made to keep your personal information confidential to the extent permitted by law. Personal information from your study records will not be released without your written permission. You will be identified by a code number known only to you and the study staff. This number – not your name – will mark all information about you. However, study records that could be used to identify you may be reviewed by the study sponsor and their authorized study monitors, local government or regulatory agencies, or the Ethics Committee/IRB. Your name will never be used in any publication or presentation about this screening process or about the research study.

All contact for follow-up visits will be handled carefully to keep your participation in this study confidential.

[insert here any country-specific legal disclosure/reporting requirements, if applicable]

RESEARCH RELATED INJURY

If you are injured as a result of being in this research study, you will be given immediate treatment for your injuries. However, you may have to pay for this care. There is no program for compensation either through this institution or the study sponsor (the US National Institutes of Health [NIH]). You will not give up any of your legal rights by signing this consent form.

PERSONS TO CONTACT FOR PROBLEMS OR QUESTIONS

For questions about this study or a research-related injury, contact:

[site insert name of the investigator or other study staff]
[site insert telephone number and physical address of above]

For questions about your rights as a research subject, contact:

[site insert name or title of person on the ETHICS Committee/Institutional Review Board (IRB) or other organization appropriate for the site]
[site insert telephone number and physical address of above]

Sample Participant Enrollment Consent Form
HPTN 062
Feasibility and Acceptability Study of an Individual-Level Behavioral Intervention for Individuals with Acute and Early HIV-Infection
DAIDS ID: 10667

US National Institute of Allergy and Infectious Diseases (NIAID)
US National Institute on Drug Abuse (NIDA)
US National Institute of Mental Health (NIMH)
US National Institutes of Health (NIH)

SIGNATURE PAGE

You understand that participation does not depend on permission to have interviews tape recorded. Please indicate what you agree to:

☐ I give my permission for the interview to be tape recorded.

☐ I do not give my permission for the interview to be tape recorded.

☐ I give my permission for the topics discussed at my post-test counseling session to be documented by the counselor.

☐ I do not give my permission for the topics discussed at my post-test counseling session to be documented by the counselor.

If you have read this consent form (or had it explained to you), all your questions have been answered, and you agree to take part in this study, please sign your name or make your mark below.

______________________	______________________________
Participant's Name (print)	Participant's Signature and Date
______________________	______________________________
Study Staff Conducting Consent Discussion (print)	Study Staff Signature and Date
______________________	______________________________
Witness's Name (print)	Witness's Signature and Date

(As appropriate)

Appendix IIB - Sample Counselor Consent Form

HPTN 062
Feasibility and Acceptability Study of an Individual-Level Behavioral Intervention Study for Individuals with Acute and Early HIV-Infection
DAIDS ID: 10667

US National Institute of Allergy and Infectious Diseases (NIAID)
US National Institute on Drug Abuse (NIDA)
US National Institute of Mental Health (NIMH)
US National Institutes of Health (NIH)

Principal Investigator: *[insert name and contact information for the site PI]*

INTRODUCTION
You are being asked to take part in the research study named above. The person in charge of this study at this site is *[insert name of PI]*. This study is sponsored by the US National Institutes of Health (NIH).

You have been asked to participate in the research study because you facilitate the enhanced HIV prevention program sessions. Before you decide whether or not you want to take part in this research study, you need to know the purpose of the study, about the possible risks and benefits, and what will be expected of you if you decide to participate. This consent form provides information about the study. The study staff will also discuss the study with you. They will answer any questions that you have. After the study has been fully explained to you, you can decide whether or not you want to participate. If you decide to participate in the study, you will be asked to sign this consent form. You will be offered a copy to keep.

VOLUNTARY PARTICIPATION
It is important that you know certain things:
- Your participation in the study is entirely voluntary.
- You may decide not to participate without impacting your employment.

PURPOSE OF THE STUDY
The purpose of the research study is to evaluate an enhanced counseling program designed to help individuals with acute and early HIV infection reduce risk behaviors for HIV transmission. The study will determine whether the counseling program is feasible to implement and acceptable to participants. This study is taking place at several sites, including at least two in Africa. Approximately 40 to 60 people will participate in the study.

STUDY PROCEDURES
To assess the program, interviews with individuals with acute HIV infection will be conducted at several time points. Interviews will also be conducted with counselors who implemented the program. You are invited to take part in this interview because you are one of these counselors. These interviews are considered research and therefore we must ask for your permission to take part.

You are being asked to take part in approximately three qualitative interviews to capture your perceptions of the acceptability and perceived impact of the intervention. During the interviews, you will be asked about your thoughts on the feasibility and acceptability of the prevention program, and what you liked and did not like about the program. You will be asked questions about your perception of the participants' experiences with the program, and on how this program can be improved. Each interview

will last about 1 to 1 ½ hours. The interviews will be tape recorded, if you permit, so a record of what was said can be maintained. You may still participate in the interviews if you choose not to have your interview tape recorded.

POTENTIAL BENEFITS
You may receive no direct benefit from this research study. However, you may find it rewarding to contribute to the development of an intervention for individuals with acute and early HIV infection aimed at reducing the risk of sexual transmission to their uninfected partners.

RISKS AND DISCOMFORTS
No risks or harms are expected to be associated with participation in this study.

REASONS WHY YOU MAY BE WITHDRAWN FROM THE STUDY WITHOUT YOUR CONSENT

- The research study is stopped or cancelled.
- Other administrative reasons.

NEW FINDINGS
You will be told of any new information learned during the course of the study that might cause you to change your mind about staying in the study.

CONFIDENTIALITY
You will be identified by a code number known only to you and the study staff. This number – not your name – will mark all information about you. However, because there are only a few staff at your site that will deliver this intervention, it is likely that researchers will know your identify and the information you share. Study records that may identify you could also be reviewed by the study sponsor and their authorized study monitors, local government or regulatory agencies, or the Ethics Committee/IRB. However, the information you discuss during the interviews will not be shared with the participants of the intervention. Also, your name will never be used in any publication or presentation about this research study.

PERSONS TO CONTACT FOR PROBLEMS OR QUESTIONS

For questions about this study contact:

[site insert name of the investigator or other study staff]
[site insert telephone number and physical address of above]

For questions about your rights as a research subject, contact:

[site insert name or title of person on the Ethics Committee/ Institutional Review Board (IRB) or other organization appropriate for the site]
[site insert telephone number and physical address of above]

Sample Counselor Consent Form
HPTN 062
Feasibility and Acceptability Study of an Individual-Level Behavioral Intervention for Individuals with Acute and Early HIV-Infection
DAIDS ID: 10667

US National Institute of Allergy and Infectious Diseases (NIAID)
US National Institute on Drug Abuse (NIDA)
US National Institute of Mental Health (NIMH)
US National Institutes of Health (NIH)

SIGNATURE PAGE

You understand that participation does not depend on permission to have interviews tape recorded. Please indicate what you agree to:

☐ I give my permission for the interview to be tape recorded.

☐ I do not give my permission for the interview to be tape recorded.

If you have read this consent form (or had it explained to you), all your questions have been answered, and you agree to take part in this study, please sign your name below.

______________________	____________________________________
Clinician/Counselor's Name (print)	Clinician/Counselor's Signature and Date
______________________	____________________________________
Study Staff Conducting Consent Discussion (print)	Study Staff Signature and Date
